# Custom-Made Poly(urethane) Coatings Improve the Mechanical Properties of Bioactive Glass Scaffolds Designed for Bone Tissue Engineering

**DOI:** 10.3390/polym14010151

**Published:** 2021-12-31

**Authors:** Monica Boffito, Lucia Servello, Marcela Arango-Ospina, Serena Miglietta, Martina Tortorici, Susanna Sartori, Gianluca Ciardelli, Aldo R. Boccaccini

**Affiliations:** 1 Department of Mechanical and Aerospace Engineering, Politecnico di Torino, Corso Duca degli Abruzzi 24, 10129 Turin, Italy; lucia.servello@polito.it (L.S.); serena.miglietta@studenti.polito.it (S.M.); martina.tortorici@outlook.com (M.T.); susanna.sartori@polito.it (S.S.); gianluca.ciardelli@polito.it (G.C.); 2 Institute of Biomaterials, University of Erlangen-Nuremberg, Cauerstr. 6, 91058 Erlangen, Germany; marcela.arango@fau.de; 3 Julius Wolff Institut, Charité—Universitätsmedizin Berlin, Augustenburger Platz 1, 13353 Berlin, Germany

**Keywords:** bioactive glass, poly(urethane)s, dip-coating, replication method, bone tissue engineering

## Abstract

The replication method is a widely used technique to produce bioactive glass (BG) scaffolds mimicking trabecular bone. However, these scaffolds usually exhibit poor mechanical reliability and fast degradation, which can be improved by coating them with a polymer. In this work, we proposed the use of custom-made poly(urethane)s (PURs) as coating materials for 45S5 Bioglass^®^-based scaffolds. In detail, BG scaffolds were dip-coated with two PURs differing in their soft segment (poly(ε-caprolactone) or poly(ε-caprolactone)/poly(ethylene glycol) 70/30 *w*/*w*) (PCL-PUR and PCL/PEG-PUR) or PCL (control). PUR-coated scaffolds exhibited biocompatibility, high porosity (ca. 91%), and improved mechanical properties compared to BG scaffolds (2–3 fold higher compressive strength). Interestingly, in the case of PCL-PUR, compressive strength significantly increased by coating BG scaffolds with an amount of polymer approx. 40% lower compared to PCL/PEG-PUR- and PCL-coated scaffolds. On the other hand, PEG presence within PCL/PEG-PUR resulted in a fast decrease in mechanical reliability in an aqueous environment. PURs represent promising coating materials for BG scaffolds, with the additional pros of being *ad-hoc* customized in their physico-chemical properties. Moreover, PUR-based coatings exhibited high adherence to the BG surface, probably because of the formation of hydrogen bonds between PUR N-H groups and BG surface functionalities, which were not formed when PCL was used.

## 1. Introduction

Musculoskeletal diseases affect hundreds of millions of people worldwide and represent one of the leading causes of long-term pain and physical disability [1]. Furthermore, as a consequence of life expectancy increase due to health care system improvement, the population is getting older, thus increasing osteoporosis incidence [2,3]. Traditional treatment methods for promoting bone healing primarily utilize bone grafts or synthetic materials to fill the defect and provide structural support. In particular, autogenous bone represents the gold standard for bone graft surgery due to its higher osteogenic potential than both allografts and xenografts [4]. However, autograft harvest requires extra surgery, thus increasing morbidity and pain, as well as the risk of massive blood loss and sepsis [4,5,6]. On the other hand, allograft and xenograft implantation increases the risk of rejection as well as the non-negligible risk of transmission of viral pathologies [6,7,8]. In this context, Bone Tissue Engineering (BTE) approaches which aim at supporting new bone tissue growth through biomaterials, cells, and specific biomolecules (e.g., growth factors), used alone or in combination, are emerging as alternatives to traditional therapies [9]. Three-dimensional (3D) scaffolds mimicking the role of the natural extracellular matrix (ECM), thus supporting the proliferation, differentiation, and biosynthesis of cells and acting as a 3D matrix for the formation of new bone tissue, are key elements in the definition of a BTE approach. The ideal scaffold for BTE should exhibit biocompatibility, osteoconductivity, osteoproductivity, highly interconnected porosity, suitable mechanical properties to allow bone regeneration and degradability in non-toxic degradation products.

Due to their chemical similarity to the inorganic phase of bone, bioceramics (such as hydroxyapatite, calcium phosphate ceramics, etc.) and bioactive glasses have been considered eligible materials to fabricate bone scaffolds [10,11,12,13,14]. 45S5 BG is a melt-derived glass based on the SiO_2_-Na_2_O-CaO-P_2_O_5_ system and commercially available in powder form. Bioactive glasses are very attractive for the fabrication of scaffolds for BTE [12,15], because of their osteoinductive behavior, ability to bond to soft tissues as well as to hard tissues and to form a carbonated hydroxyapatite layer (HCA) upon exposure to biological fluids [12,16]. BG and other silicate-based glasses stimulate the expression of several genes of osteoblastic cells through their ionic dissolution products, like Si, Ca, and P [17]. Furthermore, they can stimulate angiogenesis, both in vitro and in vivo, and exhibit antibacterial effects during dissolution due to cation release [18]. Among the different fabrication techniques available to produce bioactive glass-based scaffolds with the required morphology for bone reconstruction, the polymer foam method (or replication method) provides matrices with a microstructure similar to that of dry human trabecular bone [19]. For instance, scaffolds of silicate, borosilicate, and borate bioactive glasses have been successfully prepared with high porosities (within the range of 60–90%) [20,21,22]. In addition, the replication method produces scaffolds with different shapes and a structure close to the ideal scaffold for BTE. However, the obtained scaffolds usually exhibit poor mechanical reliability due to the high porosity and the thermal treatment required for their fabrication, which represents a compromise between bioactivity and mechanical stability of the scaffolds [19,23]. In this scenario, polymer/bioactive glass composite scaffolds represent a valid alternative due to the possibility to tailor their various properties, such as mechanical and structural behavior, degradation kinetics, and bioactivity [24]. Hence, composites combine the advantages of polymers, such as high ductility, toughness as well as favorable formability, processability, and plasticity, and those of the glass, which provides stiffness, adequate mechanical strength, and bioactivity to the resulting material. In particular, composites based on biodegradable polymers represent an interesting solution in BTE because revision surgery is not required for the removal of the implant as newly formed bone gradually substitutes the scaffold during degradation [15,25]. A currently emerging approach consists in infiltrating ceramic or bioactive glass scaffolds with a polymer [26,27,28,29], thus better mimicking the natural bone composition (i.e., composite of a mineral and an organic phase). Moreover, the polymeric coating may help in filling existing cracks in the bioactive glass scaffold caused by the sintering process of the glass, similarly to the collagen fibers of the bone tissue that bridge cracks during fracture, thus enhancing bone fracture toughness [30]. In this regard, Bertolla et al. developed a simplified 2-dimensional finite element model to assess the contribution of the coating itself and crack infiltration on mechanical properties improvement, demonstrating that polymer infiltration within surface cracks plays the key role in scaffold strengthening upon the coating procedure [31]. It has also been reported that the polymeric coating can serve as a reservoir of drugs/biomolecules to be released in a sustained manner over time [29,32,33,34]. For what concerns the polymers used to coat BG scaffolds, in 2009, Bretcanu et al. successfully coated 45S5 BG-derived glass-ceramic scaffolds with poly(3-hydroxybutyrate), demonstrating that coating the scaffolds with a polymeric layer effectively enhances their mechanical properties, with no significant effects on their bioactivity [35]. Similar outcomes have also been reported by Hum et al., Chen et al., and Fereshteh et al. using poly(ε-caprolactone) (PCL), poly(D, L-lactic acid), and blends of PCL with zein, respectively [34,36,37]. Coatings based on natural polymers have also been explored using zein, collagen, gelatin (cross-linked or uncross-linked), and cellulose [29,36,38,39]. Westhauser and colleagues have recently reported on the osteoinductive properties of differently coated 45S5 BG scaffolds (gelatin, cross-linked gelatin, and poly(3-hydroxybutyrate-co-3-hydroxyvalerate) -PHBV-), showing that new bone tissue was detectable in all the tested groups, irrespective of the material used to make the coating [40]. However, the best bone deposition was observed in gelatin-coated scaffolds probably because a highly stable coating, like cross-linked gelatin and PHBV, could reduce interaction between BG surface and surrounding tissue and cells, thus impairing new tissue formation and cell attachment/growth in the case of synthetic polymers. Hence, an accurate balance between mechanical strength enhancement and coating degradation kinetics should be defined to impart the resulting scaffolds with improved mechanical properties, while keeping unaltered osteconductivity and osteoinductivity.

In this work, custom-made poly(urethane)s (PURs) have been explored for the first time as coating materials for 45S5 BG scaffolds. Differently from commercially available synthetic and natural polymers, poly(urethane)s offer the possibility to properly select their building blocks to provide them with the optimal degradation kinetics, mechanical strength, and wettability. Moreover, poly(urethane)s can be easily functionalized in bulk using peptide sequences as building blocks [41,42] or by introducing functional groups along their backbone to be used for biomolecule grafting in mild conditions (e.g., via carbodiimide chemistry) [43,44]. In this work, PUR highly documented chemical versatility [45] has been exploited to design two poly(urethane urea)s differing in the composition of their soft segment (poly(ε-caprolactone) (PCL) and poly(ε-caprolactone)/poly(ethylene glycol) (PCL/PEG) 70/30 *w*/*w*), which resulted in polymers of variable wettability and stability in aqueous media. The protocol for BG scaffold coating with the synthesized PURs has been optimized to maximize scaffold coating without hampering their bioactivity and structural properties. The effects of coating composition on the physico-chemical, mechanical, and morphological properties of the coated scaffolds were thoroughly investigated through scanning electron microscopy, compression tests, bioactivity tests, infrared spectroscopy, X-ray crystallography, and preliminary cytocompatibility tests. Moreover, the bonding at the interface between the polymer and the bioactive glass surface was analyzed using polymer-coated pellets instead of 3D porous scaffolds. BG scaffolds coated with a commercially available poly(ε-caprolactone) were used as a control in all characterizations.

## 2. Materials and Methods

### 2.1. Materials

Poly(ε-caprolactone) (PCL) diol ($\bar{M_{n}}$ 2000 g/mol, Acros Organics, Nidderau, Germany) and poly(ethylene glycol) (PEG, $\bar{M_{n}}$ 2000 g/mol, Sigma Aldrich, Milano, Italy) were used as macrodiols during PUR synthesis. They were dried under reduced pressure (around 150 mbar) at 100 °C for 10 h and then kept at 30 °C until the reaction took place. L-lysine ethyl ester dihydrochloride (Sigma Aldrich, Italy) was used as precursor of the chain extender and stored under reduced pressure (approx. 5 mbar) at room temperature overnight, while 1,6-hexamethylene diisocyanate (HDI, Sigma Aldrich, Italy) was distilled under reduced pressure (ca. 9·10^−2^ mbar) to remove moisture and stabilizers. Supplementary Figure S1 reports the chemical structures of PUR building blocks. The synthesis occurred in a controlled atmosphere (N_2_) and anhydrous conditions. To this aim, the glassware was completely dried at 120 °C overnight before use. 1,2-dichloroethane (DCE, Sigma Aldrich, Italy) was used as a solvent for PUR synthesis. Triethylamine (TEA) and dibutyltin dilaurate (DBTDL) were purchased from Sigma Aldrich, Italy, and used as received. All solvents were purchased from Carlo Erba Reagents, Italy in the analytical grade. The PCL used as a control was purchased from Sigma Aldrich, Italy, with a number average molecular weight of 80,000 g/mol.

Concerning scaffold production, the slurry was obtained from melt-derived 45S5 Bioglass^®^ powder with particles size of approximately 4 μm, purchased from Schott, Germany and polyvinyl alcohol (PVA, completely hydrolyzed, $\bar{M_{w}}$ 30,000 g/mol) as binder acquired from Merck KGaA, Germany. The sacrificial template was a fully reticulated polyester-based poly(urethane) foam with 45 ppi (pores per inch), purchased from Eurofoams, Germany.

### 2.2. Poly(urethane) Synthesis

The synthesis of two poly(urethane urea)s used in this work was carried out according to the procedure described in [41] and schematized in Supplementary Figure S2. Briefly, the macrodiol (PCL diol or a mixture of PCL diol and PEG at 70:30 *w*/*w*) was solubilized in DCE and the resulting solution was azeotropically anhydrified by refluxing under N_2_ over molecular sieves, for at least 8 h at 80 °C; then the diisocyanate HDI (2:1 molar ratio with respect to the macrodiol) and the catalyst DBTDL were added. The reaction occurred at 80 °C for 2.5 h to finally obtain an isocyanate-terminated prepolymer. After cooling down the system at room temperature, L-lysine ethyl ester dihydrochloride (1:1 molar ratio with respect to the macrodiol) was dissolved in anhydrous DCE and added to the prepolymer. TEA was also added to induce chain extender neutralization. The reaction occurred at room temperature for 16 h and was finally stopped by adding methanol. The synthesized poly(urethane urea) was collected through precipitation in petroleum ether at a 4:1 volume ratio with respect to the total amount of DCE used during the synthesis and dried overnight under the fume hood at room temperature. The procedure of purification was performed twice by PUR dissolution in N,N-dimethylformammide (DMF) followed by precipitation in methanol at MeOH:DMF 5:1 volume ratio for PCL-based PUR. In the case of the PUR containing both PCL and PEG blocks along its backbone, purification was conducted by precipitating the polymer solution prepared in DMF in a mixture of diethyl ether and methanol (97:3 *v*/*v*) at a 5:1 volume ratio with respect to DMF. Purified PURs were dried under the fume hood at room temperature, ground, and finally stored under vacuum.

### 2.3. Poly(urethane) Nomenclature

Hereafter the synthesized PURs will be referred to as KHC2000 and KHC2000E2000. This nomenclature is based on the nature of PUR constituent blocks. The first letter K indicates the chain extender, the second H refers to the diisocyanate, while C2000 and E2000 correspond to PCL diol and PEG with number average molecular weight of 2000 g/mol.

### 2.4. Poly(urethane) Characterization

#### 2.4.1. Attenuated Total Reflectance Fourier Transform Infrared (ATR-FTIR) Spectroscopy

PUR powder was analyzed using a Perkin-Elmer Spectrum 100 equipped with an ATR accessory (UATR KRS5) with a diamond crystal. ATR-FTIR spectra of the synthesized poly(urethane)s and the starting macrodiols were obtained at room temperature in the spectral range from 4000 to 600 cm^−1^; each spectrum was obtained as a result of 16 scans with a resolution of 4 cm^−1^ and analyzed using the Perkin-Elmer Spectrum Software.

#### 2.4.2. Size Exclusion Chromatography (SEC)

The number average molecular weight ($\bar{M_{n}}$), weight average molecular weight ($\bar{M_{w}}$) and the molecular weight distribution ($D=\frac{\bar{M_{w}\;}}{\bar{M_{n}}}$) of the custom-made poly(urethane-urea)s were estimated by Size Exclusion Chromatography (SEC) (Agilent Technologies 1200 Series, Santa Clara, CA, USA). The instrument was equipped with a Refractive Index Detector (RID) and two Waters Styragel columns (HR1 and HR4). The polymer was dissolved (2 mg/mL) in a solution of N,N-dimethylformamide (DMF HPCL grade, Carlo Erba Reagents, Italy) added with Lithium Bromide (Sigma Aldrich, Italy) at 0.1% *w*/*v* and the obtained polymeric solution was filtered using a 0.45 μm poly(tetrafluoroethylene) syringe filter (Lab Logistics Group GmbH, USA). Analyses were performed at 55 °C at a flow rate of 0.5 mL/min. The chromatography peaks were converted (Agilent ChemStation Software) into a molar mass distribution by a calibration curve obtained using nine poly(methyl methacrylate) (PMMA) standards ranging in $\bar{M_{n}}$ from 4000 to 200,000 g/mol.

#### 2.4.3. Static Contact Angle Measurements

The static contact angle was measured on each PUR in the form of thin films. The samples were prepared through the conventional solvent-casting technique by dissolving 100 mg of polymer in 1 mL of DMF and dropping the resulting solution over a rectangular glass slide, that was then left to dry under the fume hood for 2 days at room temperature. A Contact Angle Measurement Instrument CAM 200 (KSV Instrument, Ltd., Espoo, Finland) was used to measure the water contact angle using a sessile drop method in advancing mode. A distilled water drop of 5 μL was gently deposited on the surface of the polymeric films and one image was recorded and analyzed through the Attension Theta software that provides an automatic curve fitting of the drop profile based on the Young and Laplace equation. Three measurements on different areas of the sample surface were performed and results are reported as mean ± standard deviation. For a better evaluation of the hydrophilic or hydrophobic nature of the synthesized PURs, the absorption time of a deposited drop was also analyzed, taking images 1, 2, and 5 min after deposition.

#### 2.4.4. Mechanical Tensile Tests

Mechanical tests were performed on PUR and PCL films prepared by the solvent casting technique. Briefly, the polymers were solubilized in 30 mL DMF at 3% *w*/*v* and the resulting solution was poured in a glass Petri dish with 70 mm diameter and left to dry under the fume hood at room temperature for 2 days. Then, samples for mechanical tensile tests were cut with approx. 10 × 5 × 0.5 mm dimensions.

Stress-strain tests were performed using an MTS QTest/10 Elite Controller equipped with a 500 N load cell. Analyses were performed in triplicate, at room temperature, and in dry conditions. During the tests, the cross-head speed was set at 10 mm/min. The collected applied force and displacement data were then used to estimate the maximum tensile stress (maximum applied force over samples’ original cross-section), the strain (expressed as a percentage of the initial length and calculated as displacement over initial length), and Young’s modulus value (slope of the line interpolating stress-strain data with strain up to 5%, i.e., linear tract) for each analyzed polymer.

#### 2.4.5. Degradation/Dissolution Tests

Hydrolytic and enzymatic degradation tests were carried out on the two PURs and on PCL in the form of thin films produced through a film casting technique according to the protocol described in Section 2.4.4. The samples for degradation experiments were 10 mm × 10 mm in size and 20–30 mg in weight. Hydrolytic degradation was performed in Phosphate Buffered Saline (PBS) solution (pH 7.4, Sigma Aldrich, Italy), while enzymatic degradation was carried out in PBS containing Lipase from Pseudomonas Cepacia (Sigma Aldrich, Italy) at 0.1% *w*/*v*.

Each sample was first weighted (*w*_0_) and then incubated at 37 °C after an appropriate volume of degradation medium was added (300 μL of solution every 5 mg of polymer) into each vial. The degradation medium was completely refreshed every 2 to 3 days. After 1, 3, 5, 7, 14, and 21 days three films were picked out, rinsed using deionized water, freeze-dried, and weighted again (*w_t_*). Degradation/dissolution was expressed as a percentage of weight loss after immersion in the degradation medium for a predefined time interval, according to Equation (1).

(1) $$\%w_{t}{}_{loss}=\frac{w_{0}-w_{t}}{w_{0}}\;\cdot 100$$

Results are reported as average value ± standard deviation.

SEC analyses were also carried out on dried samples to estimate the change in number average molecular weight during degradation (Equation (2)).

(2) $$\%{\bar{M_{n}}}_{loss}=\frac{\bar{M_{n}}\left(t_{0}\right)-\bar{M_{n}}\left(t\right)}{\bar{M_{n}}\left(t_{0}\right)}\;\cdot 100$$

where $\bar{M_{n}}\left(t_{0}\right)$ and $\bar{M_{n}}\left(t\right)$ are the estimated number average molecular weight values before the beginning of the experiment (*t*_0_) and after *t* days of incubation in the degradation medium, respectively.

Finally, modifications of film morphology during degradation were assessed through Scanning Electron Microscopy (SEM) analyses performed on golden-coated samples (Leo 1450 MP Microscope) at 20 kV beam voltage.

### 2.5. Scaffold Fabrication

Scaffolds were fabricated according to the procedure described in [20]. Cylindrical templates with 10 mm diameter and 7 mm thickness were cut from a fully reticulated polyester-based poly(urethane) foam, washed with acetone in an ultrasonic bath (Bandelin Sonorex), squeezed, and dried in an oven at 60 °C. The slurry was prepared with 49.7 wt% of deionized water (DI-water), 0.3 wt% of the binder PVA, and 50 wt% of 45S5 BG powder. Briefly, PVA was dissolved in DI-water at 80 °C under stirring for 1 h. Then, the solution was cooled down at room temperature and the BG powder was added slowly under stirring for 1 h. The polymeric template was immersed in the slurry for around 1 min and manually retrieved, the excess slurry was squeezed out by hand, and the sample was dried in the oven at 60 °C for 1 h. The coating procedure was repeated twice for each sample. The samples were then dried at 60 °C for at least 12 h. To obtain the final scaffolds, the green bodies were heated first at 400 °C for 1 h (heating rate of 2 °C/min) to burn out the poly(urethane) template and then at 1050 °C for 2 h (heating rate of 2 °C/min) to sinter the glass and finally cooled down with natural cooling until room temperature.

### 2.6. Scaffold Coating

A dip-coating procedure was used to coat 45S5 BG scaffolds with both the synthesized PURs and PCL.

Scaffolds were coated with PCL according to the optimized protocol already reported by Fereshteh et al. [34]. Briefly, PCL was dissolved in chloroform (VWR Chemicals, France) at 1% *w*/*v* and the scaffolds were completely immersed for 2.5 min. In the case of poly(urethane)s, instead, an optimization of the coating procedure was carried out by dipping BG scaffolds in polymer solutions prepared in chloroform at 1 or 0.5 % *w*/*v* concentration for 1 min or 1 day. This optimization was carried out to finally produce polymer-coated BG scaffolds with the minimum number of clogged pores and a homogeneous coating. After the coating procedure, the scaffolds were put on a glass Petri dish, frequently moved from one spot to another to remove the liquid in excess, and finally dried under the fume hood at room temperature overnight before the application of the next coating layer. The dip-coating procedure in the optimized conditions was repeated three times on each scaffold. Hereafter, PCL-, KHC2000- and KHC2000E2000-coated scaffolds will be referred to with the following acronyms: PCL/BG, KHC2000/BG, and KHC2000E2000/BG, respectively.

### 2.7. Study of Polymer-BG Bond Strength

Pellets of BG powder were produced using an electrohydraulic press (Mauthe Maschinenbau, Germany). After pressing them, the resulting BG pellets were sintered by heating them to 1050 °C for 2 h (heating rate of 2 °C/min) followed by cooling down with natural cooling until room temperature. Pellets were coated under the same conditions described for scaffolds. The morphology of the bond between the polymers and the BG pellets was studied through SEM (Carl Zeiss Microscopy, software SmartSEM) over the cross-section. Uncoated BG pellets were also analyzed as control.

### 2.8. Scaffold Characterization

#### 2.8.1. Scaffold Morphological Characterization

The morphology of pure BG and polymer-coated BG scaffolds (with 3 polymeric layers) was first observed under a light microscope (Stemi 505, Zeiss) equipped with an Axiocam 105 color camera and through Scanning Electron Microscopy (Carl Zeiss Microscopy, software SmartSEM). SEM images were recorded on golden-coated samples and analyzed with ImageJ software to estimate the average pore size and pore size distribution. The average porosity of the scaffolds was calculated before and after the coating (%p_1_ and %p_2_, respectively), using the following formulae [37]:

(3a) $${\%p}_{1}=\left(1-\frac{w_{1}}{\rho_{\mathrm{BG}}\cdot V_{1}}\right)\cdot 100$$

(3b) $${\%p}_{2}=\left[1-\frac{\left(\frac{w_{1}}{\rho_{\mathrm{BG}}}+\frac{w_{2}-w_{1}}{\rho_{\mathrm{coat}}}\right)}{V_{2}}\right]\cdot 100$$

where V_1_ and w_1_ stand for the volume and the weight of the samples before the coating, V_2_ and w_2_ are the volume and the weight of the samples after the coating procedure, $\rho_{\mathrm{BG}}$ = 2.7 g/cm^3^ is the density of solid 45S5 BG and $\rho_{\mathrm{coat}}$ is the density of the polymer used for the coating. PCL density has been already reported by Fereshteh et al. [34], whereas that of the here-synthesized PURs was experimentally estimated (Table 1). In detail, polymer density was experimentally calculated by measuring the dimensions and the weight of dense films prepared by solvent casting.

#### 2.8.2. Scaffold Bioactivity

The protocol described by Kokubo et al. [46] was used to assess the bioactivity of the produced scaffolds. Simulated Body Fluid (SBF) was prepared by adding the reagents (Supplementary Table S1) to double demineralized water while stirring, in controlled conditions of temperature (36.5 ± 1.5 °C) (see Supplementary Information file for a detailed description of SBF preparation protocol). PCL/BG, KHC2000/BG, and KHC2000E2000/BG samples were immersed in 50 mL of SBF and incubated under slow tangential agitation at 37 °C (Inkubator 1000, Heidolph, Schwabach, Germany). SBF was refreshed twice a week and after 1, 3, 7, 14, and 21 days, three samples of each type of scaffolds were extracted from the SBF solution, rinsed gently with deionized water, and left to dry at 37 °C. Scaffold bioactivity was then assessed by SEM and ATR-FTIR spectroscopy (IRAffinity-1S Fourier Transform Infrared Spectrophotometer Shimadzu). Moreover, the crystallinity of the deposited hydroxyapatite (HA) was evaluated through X-rays diffraction (XRD) analysis (Rigaku MiniFlex600). ATR-FTIR and XRD were performed on sample powder.

#### 2.8.3. Degradation Tests

Hydrolytic degradation was studied under slow tangential agitation at 37 °C by dipping each scaffold (with an initial weight *w*_0_) in 50 mL of PBS solution. Three samples were collected from the solution after 1, 3, 7, 14, and 21 days, abundantly rinsed with DI-water, and dried at 37 °C until a constant weight was reached (*w_t_*). The percentage of weight loss was calculated according to Equation (1). The pH of PBS was also measured at each time point after sample removal.

#### 2.8.4. Mechanical Tests

The compressive mechanical properties of uncoated and polymer-coated scaffolds were measured using a Zwick/Roell Z050 mechanical tester at a crosshead speed of 5 mm/min. For pure BG scaffolds and PCL-coated samples, a cell load with a capacity of 50 N was used, meanwhile for PUR-coated matrices a 1 kN cell load was required. During the test, an increasing load was applied on the scaffold until the compressive strain reached 75%. The compressive strength was determined from the maximum load of the obtained stress-strain curve. The work of fracture (*W*), which is related to the energy required to deform a sample up to a certain deformation, was calculated from the area under the stress-strain curve at a given strain (before densification). To better simulate the in vivo environment, the scaffolds were also tested in wet conditions [47]. To this aim, the samples were soaked in PBS for 30 min before the compressive strength test. Five samples for each condition were tested and the results are presented as the average value and standard deviation. Mechanical tests were also performed on BG and polymer-coated scaffolds after 3, 7, 14, and 21 days of immersion in SBF, to assess their mechanical behavior during concurrent degradation and HA deposition. At each time point, the change in compressive strength (*σ*_residual_) was calculated according to Equation (4).

(4) $$\sigma_{\mathrm{residual}}\left(\%\right)=\frac{\sigma_{t}}{\sigma_{0}}\cdot 100$$

where $\sigma_{0}$ represents the initial compressive strength, while $\sigma_{t}$ is the measured compressive strength after immersion in SBF for *t* days.

### 2.9. Biological Tests

#### 2.9.1. Preparation of Scaffolds

Before cell seeding, the scaffolds were cleaned by consecutive soaking in Sodium Dodecyl Sulfate (SDS, Carl Roth GmbH, Ludwigshafen, Germany) and Extran (Merck, Germany) solutions (2% *w*/*v* and 5% *w*/*v*, respectively) for 5 min. After drying, BG scaffolds were sterilized at 160 °C for 2 h in a furnace, meanwhile, polymer-coated scaffolds were put under UV light for 3 h. BG, KHC2000/BG, KHC2000E2000/BG, and PCL/BG scaffolds were then preconditioned in Dulbecco’s Modified Eagle Medium (DMEM, Thermo Fisher, Karlsruhe, Germany) without phenol red at 37 °C, 2% O_2,_ and 10% CO_2_ (Galaxy 48 R, Fredericton, NB, Canada) for six days before cell seeding.

#### 2.9.2. Cell Seeding and Cultivation

The osteosarcoma cell line MG-63 (Sigma Aldrich, Germany) was used to assess scaffold biocompatibility. Cells were cultured in DMEM (Thermo Fisher, Germany) containing 10% *v*/*v* fetal bovine serum (FBS, Sigma Aldrich, Germany) and 1% *v*/*v* penicillin/streptomycin (Gibco, Germany), at 37 °C, 5% CO_2_ and 95% humidity (Galaxy 170 R, New Brunswick). After the preconditioning period, the samples were placed in a 24-well plate and seeded with 10^5^ cells using a drop-on method. Cells seeded and cultivated on a 24 well-plate were used as a positive control. Complete cell culture refresh was performed every day.

#### 2.9.3. Cell Viability and Staining

Cell viability was assessed after two days of cell culture using a cell counting kit (Cell Counting Kit—8, Sigma Aldrich). Briefly, the samples were moved into a new 24-well plate and a fresh medium containing 3% *v*/*v* WST (a water-soluble tetrazolium salt) was added. The samples were incubated for 3 h and then absorbance at 450 nm was read with a microplate reader (PHOmo, Autobio Labtec Instruments Co., Ltd., Zhengzhou, China). Cell viability (*%_viability_*) was then estimated according to Equation (5).

(5) $${\%}_{viability}=\frac{OD_{composite}-OD_{WST}}{OD_{BG}-OD_{WST}}\cdot 100$$

where *OD_composite_*, *OD_WST_*, and *OD_BG_* are the measured absorbance values at 450 nm of composite samples, *WST* (blank) and *BG* (positive control), respectively.

Cell actin filaments and nuclei were also visualized using Rhodamine Phalloidin (Thermo Fisher, Germany) and DAPI (4′,6-diamidino-2-phenylindole, dilactate, Invitrogen, MA, USA) according to supplier instructions. Images of the stained cells were taken through a fluorescence microscope (Axio Scope A.1, Carl Zeiss Microimaging GmbH, Germany).

### 2.10. Statistical Analysis

Results are reported as mean ± standard deviation. One-way analysis of variance (ANOVA) was performed using GraphPad Prism version 5.00 for Windows (GraphPad Software, CA, USA; www.graphpad.com, last access date 15 December 2021). The pairwise comparison of the means was obtained with Bonferroni’s test (post hoc comparison). The statistical significance of the results was defined according to Boffito et al. [48].

## 3. Results and Discussion

### 3.1. Poly(urethane) Characterization

#### 3.1.1. Chemical Characterization

ATR-FTIR spectroscopy demonstrated the successful synthesis of both KHC2000 and KHC2000E2000 (Supplementary Figure S3). Newly formed urethane/urea groups showed absorbance at 1620 cm^−1^ (C=O stretching (amide I)), 1560 cm^−1^ (simultaneous N-H bending, and C-N stretching vibrations (amide II)) and in the region 3340–3360 cm^−1^ (N-H stretching). The inclusion of PCL blocks in both KHC2000 and KHC2000E2000 was proved by the presence of a peak at ca. 1723 cm^−1^ due to the stretching vibration of PCL carbonyl groups (C=O) and the absorption band at 1160 cm^−1^ due to the stretching of the C-O-C linkages [41]. KHC2000E2000 ATR-FTIR spectrum also showed absorbance at 1099 cm^−1^ due to the vibration of the CH_2_-O-CH_2_ linkages of PEG blocks. In both PURs, CH_2_ asymmetric and symmetric stretching vibrations were located at 2938 and 2865 cm^−1^, respectively. The absence of absorbance at 2260 cm^−1^ indicated that no unreacted isocyanate groups were still present in both KHC2000 and KHC2000E2000 [49]. Moreover, no peaks due to residual DMF were observed near 1673 cm^−1^ [50], suggesting a successful and complete drying of the materials.

KHC2000 exhibited a number average molecular weight ($\bar{M_{n}}$) of 68,600 g/mol and a polydispersity index (D) of 1.8, meanwhile KHC2000E2000 presented $\bar{M_{n}}$ of 48,400 g/mol and a polydispersity index of 2.1. The low polydispersity indices designated a narrow distribution of the molecular weights and a good control of the polymerization process.

#### 3.1.2. Contact Angle Measurements

KHC2000 and KHC2000E2000 exhibited average contact angle values of 123 ± 2° and 77.5 ± 0.5°, respectively. These results are in accordance with the hydrophobic nature of the PCL block [51,52]. On the other hand, the presence of PEG blocks in KHC2000E2000 resulted in a significantly higher hydrophilic nature of this polymer with respect to KHC2000 (*p* < 0.001). The difference between the two poly(urethane)s was also evident by analyzing the water uptake after drop deposition on the polymeric surface (Supplementary Figure S4). As a matter of fact, KHC2000 exhibited a slow change of the contact angle within the first 5 min after drop deposition on its surface with a reduction in contact angle of approximately 7°. On the other hand, the behavior of KHC2000E2000 was completely opposite, with a reduction in contact angle of about 40° within the 5 min of observation, thus confirming its higher wettability.

#### 3.1.3. Mechanical Characterization

**Figure 1:**
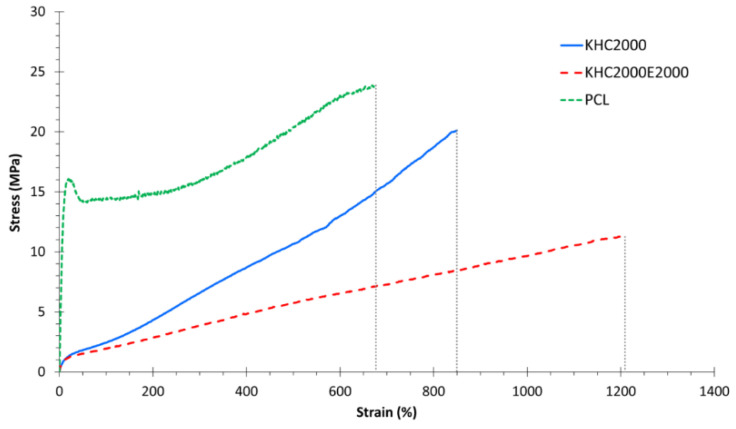
Stress-strain curves of PCL (green, dotted line), KHC2000 (blue, continuous line) and KHC2000E2000 (red, dashed line).

The mechanical characterization of PCL and the synthesized poly(urethane)s was performed by uniaxial stress-strain tests. In accordance with our previous data on poly(urethane)s with similar composition [41,42], the registered stress-strain curves revealed an elastomeric behavior for both the synthesized PURs (Figure 1). Figure 1 also reports the typical stress-strain curve of PCL for comparison.

Both KHC2000 and KHC2000E2000 showed a typical elastic behavior up to approx. 20% of strain, followed by a plastic deformation characterized by a sharp increase in stress, probably due to a strain-induced crystallization of the macrodiols [53]. Table 2 summarizes Young’s Modulus, Stress at break, and Strain at break data of PCL, KHC2000, and KHC2000E2000.

The introduction of PEG moieties along the PUR backbone turned out to affect the mechanical properties of the resulting materials, as already reported by Silvestri et al. [42]. Although PEG introduction induced a slight, not significant decrease in the stiffness and stress at break of the resulting PUR, it detectably enhanced polymer strain at break (0.0001 < *p* < 0.001).

#### 3.1.4. Degradation/Dissolution Tests

KHC2000 and KHC2000E2000 dense films were characterized in terms of their degradability through hydrolytic degradation tests in physiological-like conditions, i.e., at 37 °C in phosphate-buffered saline at pH 7.4 (PBS). Accelerated enzymatic degradation tests were also conducted adding Lipase from Pseudomonas Cepacia (0.1% *w*/*v*) to PBS. Dense films prepared starting from the commercial PCL were also characterized.

**Figure 2:**
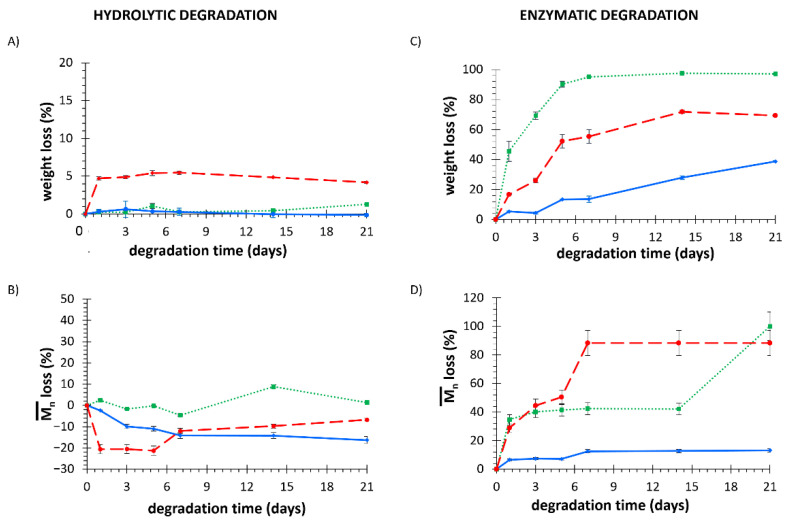
Weight loss profile of KHC2000 (blue, continuous line), PCL (green, dotted line), and KHC2000E2000 (red, dashed line) during hydrolytic (**A**) and enzymatic (**C**) degradation at 37 °C. Number average molecular weight loss profile for KHC2000 (blue, continuous line), PCL (green, dotted line), and KHC2000E2000 (red, dashed line) during hydrolytic (**B**) and enzymatic (**D**) degradation at 37 °C.

The trend of weight loss (%) during hydrolytic degradation of KHC2000, KHC2000E2000, and PCL films in PBS is reported in Figure 2A.

After 21 days of incubation in PBS, commercial PCL exhibited a percentage of weight loss of 1.3 ± 0.1%. The absence of evident changes in PCL mass can be ascribed to its high hydrophobicity and semi-crystalline nature, according to already reported data in the literature [54,55]. Similarly, KHC2000 PUR exhibited almost no degradation after immersion in PBS for 21 days. On the other hand, due to the presence of PEG as building block, the PUR KHC2000E2000 showed a much faster loss in mass than the other investigated materials. In fact, after 1 day incubation in PBS, a weight loss of 4.7 ± 0.7% was measured for KHC2000E2000, whereas PCL and KHC2000 did not show any weight change (*p* < 0.001). This phenomenon is most likely due to the destabilization/dissolution of KHC2000E2000 rather than to real chemical degradation. To better understand the degradation mechanism, the residual polymeric films after hydrolytic degradation were also analyzed through SEC and the loss in number average molecular weight (expressed as a percentage with respect to its initial value) has been plotted as a function of time in Figure 2B, meanwhile, Supplementary Figure S5A reports the trends of normalized refractive index (RID) signal against retention time for each analyzed sample. Regarding PCL, number average molecular weight did not show significant changes during hydrolytic degradation up to 21 days, being the characteristic error of SEC analysis in the order of 10% [56]. As a matter of fact, RID curves (Supplementary Figure S5A) seemed to slightly shift randomly around a central value. A more evident change in $\bar{M_{n}}$ and molecular weight distribution was observed for KHC2000 films, which number average molecular weight increased by 16% after 21 days of incubation in PBS at 37 °C. This unexpected increase in molecular weight can be explained by the trends of RID curves: the RID curve of native KHC2000 (0d) presented a large distribution, which became increasingly narrow with increasing incubation time, as a consequence of the progressive solubilization of short chains in the degradation medium. As a matter of fact, the polydispersity index slightly decreased after immersion in PBS for 3 weeks from 1.8 to 1.6, proving that the observed increase in $\bar{M_{n}}$ can be correlated to a narrower molecular weight distribution. Similarly, in KHC2000E2000 PUR the presence of PEG segments arranged randomly in the polymeric chains increased polymer wettability and promoted its progressive solubilization, starting from shorter chains. In fact, after only 1 day of incubation in PBS at 37 °C, the molecular weight of KHC2000E2000 increased by approx. 20% due to dissolution of short chains. Then, from day 5 to day 7 incubation, $\bar{M_{n}}$ slightly decreased approx. 7%. Although this decrease in molecular weight fell within the typical range of SEC error, the observed change in the trend of $\bar{M_{n}}$ loss, which was absent in KHC2000, could be ascribed to the onset of other destabilization phenomena affecting the PUR, such as the oxidation of its ethylene oxide moieties.

Figure 2C reports the trend of weight loss (%) of KHC2000, KHC2000E2000, and PCL films during enzymatic degradation in PBS added with Lipase from Pseudomonas Cepacia at 37 °C. PCL films showed the fastest degradation kinetics (*p* < 0.001), reaching a weight loss of 97.1 ± 0.4% after 21 days of incubation. This behavior was confirmed by literature data [54,57,58] and might be ascribed to the presence of a high number of ester bonds that are subjected to lipase-mediated hydrolysis. Indeed, the theoretical number of ester (ES) bonds in the PCL used in this work was estimated to be approx. 700 units per polymer chain (calculated as the ratio between PCL number average molecular weight and the number average molecular weight of its repeating unit (114.14 g/mol)). On the other hand, after 21 days of accelerated enzymatic degradation, KHC2000 and KHC2000E2000 samples exhibited a weight loss of 38.7 ± 0.6% and 69.3 ± 0.8%, respectively. The lower weight loss observed for KHC2000 and KHC2000E2000 compared to commercial PCL could be correlated to the lower number of ES bonds they contained. In fact, by adapting the previous formula to PURs, KHC2000 and KHC2000E2000 were estimated to theoretically possess around 27 and 13 ester bonds per polymer chain, respectively. However, based on hydrolytic degradation data, in the case of KHC2000E2000 other destabilization phenomena were hypothesized to occur during incubation in aqueous media (i.e., polymer chain dissolution and PEG oxidation). Hence, although KHC2000E2000 was estimated to possess a lower number of ES bonds compared to KHC2000, it exhibited significantly higher weight loss with respect to KHC2000 at each tested degradation time (*p <* 0.1 on day 1 and then *p* < 0.001). As a consequence of ester bond hydrolytic cleavage mediated by lipase, all the samples showed a progressive decrease in molecular weight during enzymatic degradation (Figure 2D). Supplementary Figure S5B, instead, reports the trends of the normalized RID signal as a function of retention time for each analyzed sample. Commercial PCL lost approx. 40% of its initial number average molecular weight after a 1 day incubation in PBS in the presence of lipase; as a matter of fact, its RID curve (Supplementary Figure S5B) clearly shifted towards a lower molecular weight [59,60]. Afterward, the percentage of molecular weight lost remained almost constant, probably because of the co-presence of two opposite phenomena: lipase enzymatic activity that progressively cleaves ES bonds leading to a decrease in molecular weight and the progressive loss of chains short enough to be dissolved in the medium resulting in an overall increase in the molecular weight. The KHC2000 RID curve also slightly shifted towards lower molecular weight after 1 day incubation, but its loss in $\bar{M_{n}}$ was significantly lower compared to PCL (5.4 ± 0.3% vs. 45.5 ± 6.7%), which is in accordance with the previously discussed weight loss data. However, differently from PCL, KHC2000 lost approx. 13% of its initial $\bar{M_{n}}$ after 21 days of enzymatic degradation, reaching a final $\bar{M_{n}}$ of 60,700 g/mol. In contrast with KHC2000, KHC2000E2000 PUR presented a higher variation in terms of molecular weight, as highlighted by the trend of its RID signals reported in Supplementary Figure S5B. KHC2000E2000 lost almost 40% of its initial molecular weight after 5 days of incubation and reached a plateau after 7 days, corresponding to a $\bar{M_{n}}$ loss of approx. 90% (final $\bar{M_{n}}$ of 5600 g/mol). This behavior was confirmed by the trend of RID curves, which moved towards higher elution time (i.e., lower molecular weight). Moreover, a multimodal distribution of the RID signal was observed for this PUR after 7 days of immersion in PBS added with lipase, suggesting the formation of new species with lower molecular weight but insoluble in the surrounding aqueous medium within the investigated observation time [59]. This drastic change in the RID signal trend further corroborated the previously hypothesized complex degradation mechanism of KHC2000E2000.

**Figure 3:**
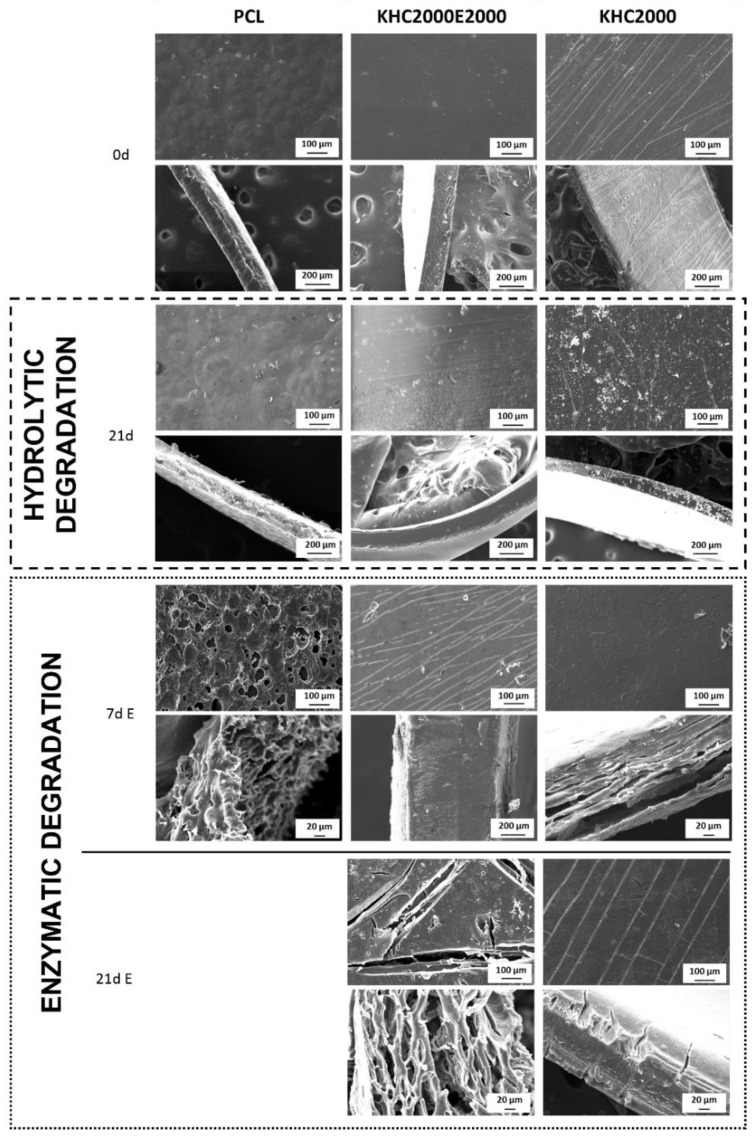
SEM micrographs of the surface and cross-section of PCL, KHC2000, and KHC2000E2000 films before degradation onset (0d) and after hydrolytic degradation in PBS at 37 °C for 21 days and enzymatic degradation in PBS added with lipase at 37 °C for 7 and 21 days (on day 21 PCL sample was not analyzed due to its complete degradation).

SEM images of the surface and the cross-section of PCL, KHC2000, and KCH2000E2000 films before (0d) and after hydrolytic and enzymatic degradation are reported in Figure 3.

In accordance with previously discussed data, no changes in surface and cross-section morphologies were observed in PCL samples after 21 days of immersion in PBS. Conversely, enzymatic degradation led to drastic changes in sample surface and cross-section. Due to the complete degradation observed on day 21, SEM analysis was performed on PCL films subjected to 7 days incubation in PBS added with lipase: due to the enzymatic activity of lipase, the film appeared to be worn out and thinner compared to the control sample due to the progressive surface erosion which characterizes this kind of degradation mechanism [44,60]. SEM images of KHC2000 films confirmed its higher resistance to degradation compared to the other investigated materials; indeed, it did not exhibit any morphological change after 21 days of hydrolytic degradation, whereas it presented some cracks on the surface after 3 weeks of immersion in PBS containing lipase. Finally, with regard to KHC2000E2000 PUR, in accordance with previous hypotheses, a form of bulk degradation was observed through SEM imaging, probably due to the presence of PEG moieties that favored the permeation of the degradation medium through sample thickness and the progressive destabilization/dissolution of water-soluble polymer chains.

### 3.2. Pure BG Scaffolds Characterization

The weight, diameter, height, porosity, and pore size of pure BG scaffolds produced through the foam replica technique are summarized in Table 3. The obtained values are typical for scaffolds produced with this technique [20].

**Figure 4:**
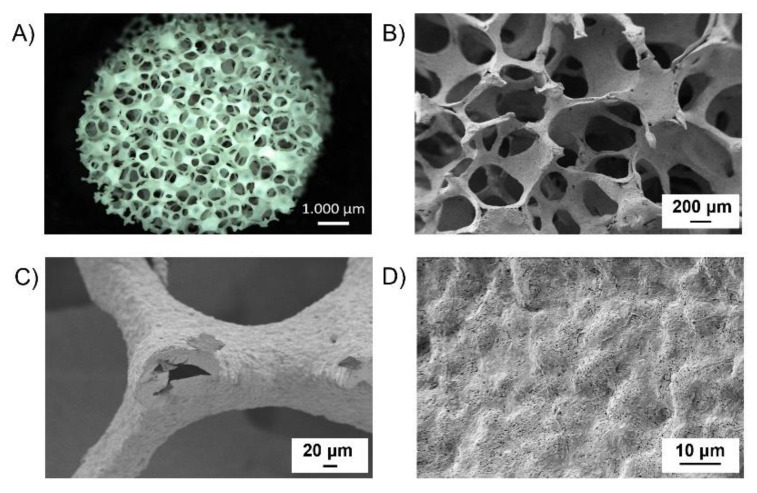
(**A**) Light microscope image and SEM micrographs at (**B**) 70×, (**C**) 500× and (**D**) 3.5k× of BG scaffolds.

Pore size turned out to be quite homogeneously distributed in the range 200–500 μm (Supplementary Figure S6), which makes the fabricated scaffolds suitable for bone tissue engineering applications, in accordance with many in vitro studies that reported a pore size greater than 200 μm to be required to allow osteoconduction [61]. A macroscopic view of the structure of the sintered scaffolds is given in Figure 4A, meanwhile, Figure 4B–D shows the macro- and micro-structure of as-fabricated BG scaffolds at different magnifications.

In agreement with the adopted fabrication technique, a highly interconnected pore structure was obtained. At high magnification (Figure 4D) the presence of sintered particles as constituents of the structure can also be appreciated. The hollow nature of the structures showed in Figure 4C can be associated with the burning out of the sacrificial poly(urethane) foam. In general, during the heating phase at 1050 °C, the densification of the scaffold structure takes place by the sintering of the glass particles and partially crystallization of the glass occurs [62]. In detail, the here-fabricated porous structures showed a slightly higher shrinkage in height (SH) than in diameter (SD) (0.27 vs. 0.22), according to Bretcanau et al. [63].

### 3.3. Optimization of BG Scaffold Coating with PURs

**Figure 5:**
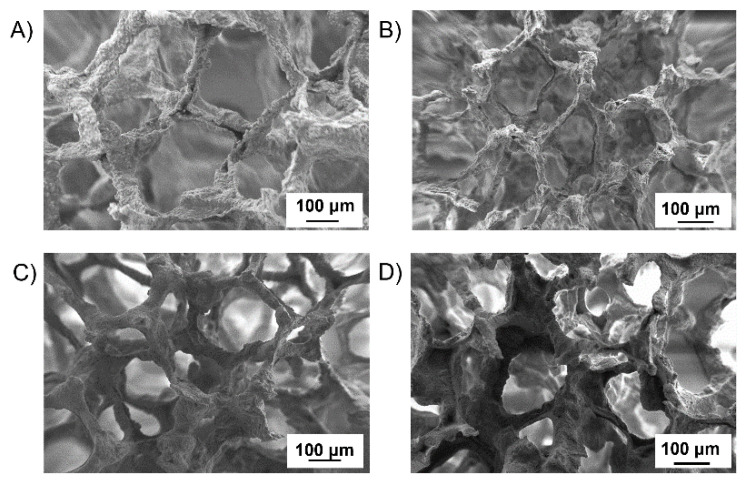
SEM images of a BG scaffold coated in a KHC2000E2000 solution in chloroform with 0.5% *w*/*v* (**A**,**B**) or 1% *w*/*v* (**C**,**D**) concentration through a 1-min (**A**,**C**) or a 1-day (**B**,**D**) dip-coating procedure.

Whereas for commercial PCL the protocol for BG scaffold coating has already been reported by [34], in the case of the PURs an optimization was required to define the best coating procedure for porous BG structures. To this aim, polymer-coated BG scaffolds according to all the investigated conditions were analyzed by SEM to select the best combination of PUR solution concentration and scaffold immersion time that led to samples with an open porous structure (i.e., poor pore-clogging) and homogenous coating. A non-adherent and non-homogeneous coating was obtained by dipping the scaffolds for 1 min (Figure 5A) or 1 day (Figure 5B) in a 0.5% *w*/*v* concentrated solution of KHC2000E2000 in chloroform, suggesting that this polymer concentration was not high enough to allow a uniform coating, filling the hollows and the cracks of the structures. With increasing KHC2000E2000 solution concentration to 1% *w*/*v* a better coating was achieved, as shown in Figure 5C,D. However, coated scaffolds obtained through a 1-day dip-coating procedure in a 1% *w*/*v* concentrated KHC2000E2000 solution in chloroform revealed some clogged pores (Figure 5D). Hence, the best coating was obtained by dipping the scaffold in a KHC2000E2000 solution in chloroform with 1% *w*/*v* concentration for 1 min (Figure 5C). Indeed, in these conditions, the coating resulted to be adherent to the scaffold and homogenous.

**Figure 6:**
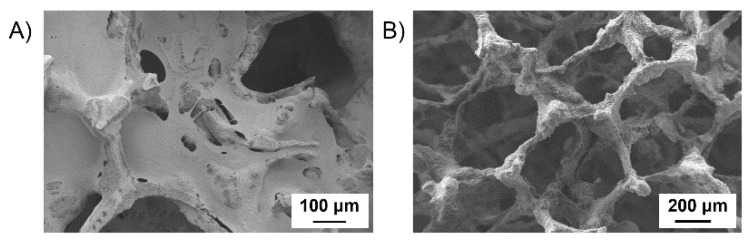
SEM images of a BG scaffold coated in a KHC2000 solution in chloroform with 1% *w*/*v* (**A**) or 0.5% *w*/*v* (**B**) concentration through a 1-min dip-coating procedure.

Considering that the 1-min dip-coating procedure in a polymer solution at 1% *w*/*v* concentration resulted to be the best condition to coat BG scaffolds with KHC2000E2000, the same procedure was adopted to produce KHC2000/BG scaffolds. However, KHC2000-coated BG scaffolds according to this protocol reported many clogged pores, probably because of the higher viscosity of 1% *w*/*v* KHC2000 solution compared to KHC2000E2000-based one with the same concentration as a consequence of its higher molecular weight (Figure 6A). Hence, KHC2000 solution concentration was decreased to 0.5% *w*/*v*. Composites produced with a 1-min dip-coating procedure of BG scaffolds in a 0.5% *w*/*v* concentrated KHC2000 solution exhibited a highly porous structure with no clogged pores (Figure 6B).

### 3.4. Investigation of Polymer/BG Adhesion

**Figure 7:**
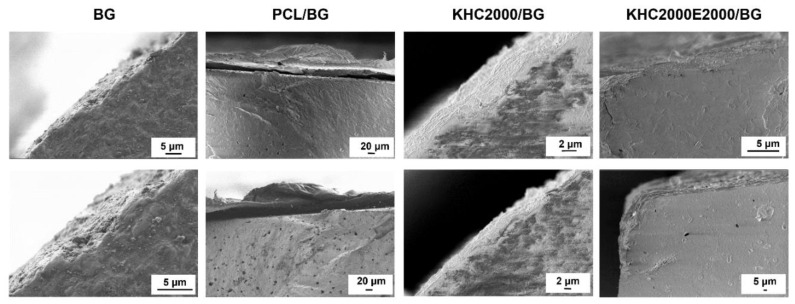
SEM images of the cross-sections of PCL-, KHC2000- and KHC2000E2000-coated pellets. SEM images of BG pellets are also reported as a control condition.

To study the interactions occurring between each type of polymer and the scaffold, 45S5 BG pellets were produced and coated with the same procedure previously optimized for scaffolds. Then, the cross-sections of the coated pellets and BG pellets as such (control) were analyzed by SEM. The obtained images allowed a qualitative analysis of the interactions occurring between the polymer and the 45S5 BG pellet (Figure 7). The polymeric coating obtained with PCL appeared as a thin poorly adherent film, whereas pellets coated with either KHC2000 or KHC2000E2000 revealed a more adherent coating. This different behavior could be ascribed to the formation of H-bonds between the N-H groups of urethane and urea bonds and the Si-O-Si groups exposed on the 45S5 BG pellet surface.

### 3.5. Characterization of Polymer-Coated BG Scaffolds

#### 3.5.1. Morphological Analysis

**Figure 8:**
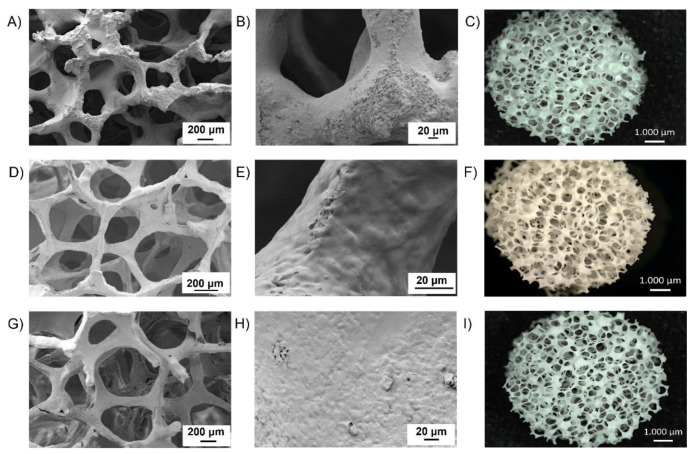
SEM micrographs and light microscope image (top-view) of PCL/BG scaffolds (**A**–**C**), KHC2000/BG scaffolds (**D**–**F**), and KHC2000E2000/BG scaffolds (**G**–**I**).

Detailed morphological characterization was carried out on composite scaffolds with 3 polymeric coating layers (Figure 8). PCL/BG composite scaffolds revealed some clogged pores, clearly visible in Figure 8C, and the coating seemed to be not homogeneous, with portions of the structure not covered with the polymer (Figure 8B). Conversely, KHC2000/BG scaffolds exhibited a highly porous structure with no clogged pores (Figure 8D). Compared to PCL, the PUR led to a more homogenous coating and the polymer better penetrated into the scaffolds’ cracks resulting from the sintering process. Interestingly, the coating obtained with KHC2000 resulted to be more homogeneous than that obtained with PCL albeit BG scaffolds were dipped in a less concentrated polymer solution for a shorter dipping time. Similarly to KHC2000/BG samples, also KHC2000E2000/BG composites showed a homogenous and adherent coating. The better coating achieved with PURs compared to PCL can be correlated with the enhanced chemical interactions occurring between PUR chains and 45S5 BG which result in a stronger bonding of the polymer to the underlying inorganic phase, as previously observed with 45S5 BG dense pellets.

Despite the presence of a small percentage of clogged pores, the characteristic open porosity of BG scaffolds was well maintained in the samples coated with either KHC2000 or KHC2000E2000, whereas a slight decrease in porosity was measured for PCL-coated scaffolds (Table 4). Additionally, the amount of KHC2000 covering the scaffold was lower with respect to the amount of both PCL and KHC2000E2000 due to the lower concentration of its polymeric solution (Table 4). On the other hand, no differences were observed between PCL- and KHC2000E2000-coated scaffolds despite the different dipping time used in their preparation. This result further corroborated the role exerted by hydrogen-bond formation between the 45S5 BG and the poly(urethane) in favoring the achievement of the same coating yield within a shorter dipping time; additionally, hydrogen-bonds were also responsible for the formation of a more adherent and homogeneous polymeric coating, as observed also for 45S5 BG dense pellets.

#### 3.5.2. Bioactivity Tests

**Figure 9:**
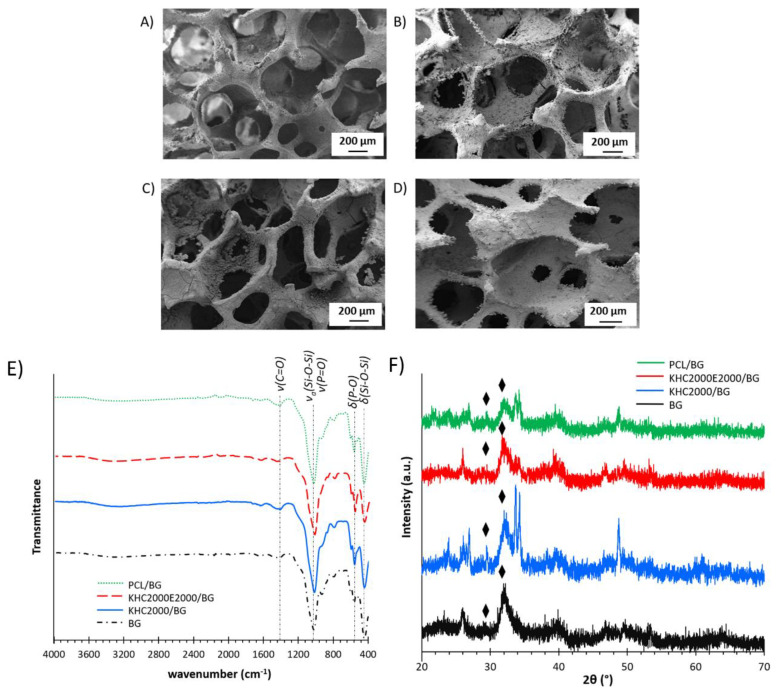
SEM images of (**A**) BG scaffold, (**B**) PCL/BG scaffold, (**C**) KHC2000/BG scaffold, (**D**) KHC2000E2000/BG scaffold after 21 days immersion in SBF. ATR-FTIR (**E**) and XRD (**F**) spectra of BG, PCL/BG, KHC2000/BG, and KHC2000E2000/BG scaffolds after 21 days immersion in SBF. The symbol identifies the characteristic peaks of the deposited crystalline HA at 2Θ values of 26° and 32°.

In vitro bioactivity of the composite scaffolds was investigated after sample incubation in SBF for different time intervals (1d, 3d, 7d, 14d, and 21d). After 21 days of incubation in SBF, the surface of both polymer-coated and uncoated samples was covered with a deposited hydroxyapatite layer as shown in Figure 9A–D. The ATR-FTIR and XRD spectra of BG, PCL/BG, KHC2000/BG and KHC2000E2000/BG samples after 21 days immersion in SBF are reported in Figure 9E,F. A detailed analysis of the changes occurring in the ATR-FTIR and XRD spectra of BG scaffolds during incubation in SBF is also reported in the Supplementary Information file (Supplementary Figure S7).

Figure 9E compares the ATR-FTIR spectra of BG, PCL/BG, KHC2000/BG, and KHC2000E2000/BG scaffolds after 21 days of immersion in SBF. The characteristic peaks of the deposited HCA layer (i.e., C = O stretching at 1418 cm^−1^, P = O stretching and bending at 1100 cm^−1^ and ca. 570 cm^−1^, respectively) were also present in the ATR-FTIR spectra of polymer-coated scaffolds, proving that the polymer coating did not inhibit BG bioactivity, as also proved by SEM imaging (Figure 9A–D). HCA layer deposition during immersion in SBF was further investigated by XRD analyses. Figure 9F compares the XRD spectra of BG, PCL/BG, KHC2000/BG, and KHC2000E2000/BG scaffolds after 21 days of incubation in SBF. The polymer coating turned out to slightly protect the crystalline phase from degradation, decreasing the transformation kinetics of the crystalline phase into the amorphous one. As a matter of fact, in the spectra of coated scaffolds, the major peak at 34° was still evident upon sample incubation in SBF for 21 days. On the other hand, crystalline HA was also deposited on polymer-coated scaffolds as demonstrated by the presence of the peaks at 26° and 32° in their XRD spectra [64].

Hence, the characteristic bioactivity of 45S5 BG scaffolds was retained in the composite samples, although the coating slightly delayed HA deposition and BG crystalline phase degradation. The well-maintained bioactivity was probably due to some small uncoated regions on the surface of the scaffolds, resulting from an incomplete coating during the dipping procedure or the progressive degradation/dissolution/detachment of the coating during immersion in aqueous media. In these regions the ions exchange between SBF and 45S5 BG occurred, thus activating the bioactive mechanism leading to the nucleation of HA crystals [35].

#### 3.5.3. Degradation Tests

**Figure 10:**
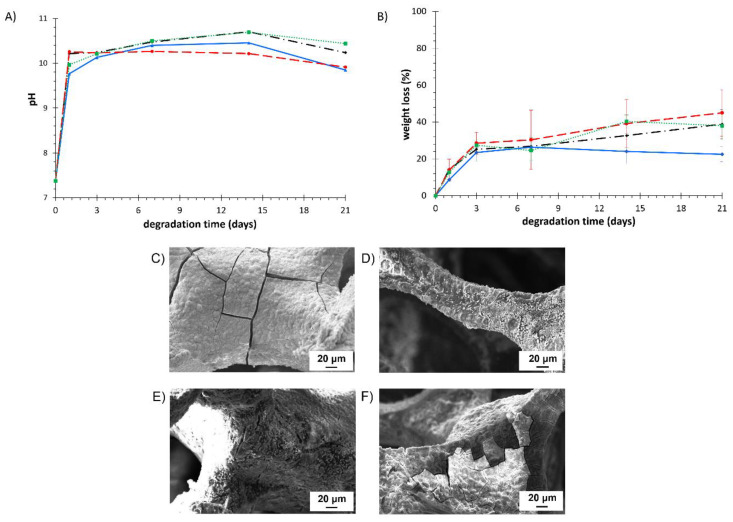
(**A**) pH variation of the PBS containing pure BG (black, dashed-dotted line), PCL/BG (green, dotted line), KHC2000/BG (blue, continuous line), KHC2000E2000/BG (red, dashed line) scaffolds during hydrolytic degradation tests. (**B**) Weight loss profile of pure BG (black, dashed-dotted line), PCL/BG (green, dotted line), KHC2000/BG (blue, continuous line), KHC2000E2000/BG (red, dashed line) scaffolds during hydrolytic degradation in PBS. SEM micrographs of (**C**) BG, (**D**) PCL/BG, (**E**) KHC2000/BG and (**F**) KHC2000E2000/BG scaffolds after 21 days immersion in PBS.

Degradation tests were carried out by soaking BG and polymer-coated BG scaffolds in PBS for 1, 3, 7, 14, and 21 days. PBS pH and sample weight loss were measured at each time point and results are summarized in Figure 10A,B. As a consequence of the well-known burst release of 45S5 BG, pH quickly increased after only 1 day of sample immersion in PBS, with a typical value in the range of 9.7–10.2. No significant effects on pH variation were observed in polymer-coated samples.

Pure BG scaffolds showed a gradually increasing weight loss, which reached 38.9 ± 8.0% after 21 days of immersion in PBS. As extensively reported in literature, the mechanism of degradation of 45S5 BG-based scaffolds is based on the progressive dissolution of the amorphous and crystalline phases and the transformation into an amorphous calcium-phosphate layer [65]. This dissolution is dependent on many factors, such as glass composition, chemical and morphological characteristics of the surface, composition of the solution in which the scaffold is immersed, and crystallinity. Coating with PCL did not allow a controlled degradation kinetics: PCL/BG samples presented almost the same behavior of uncoated BG scaffolds, probably as a consequence of the not homogeneous coating. On the contrary, KHC2000/BG scaffolds exhibited slower degradation kinetics compared to BG scaffolds, starting from 7 days incubation. The absence of differences between KHC2000/BG and pure BG scaffolds up to 7 days incubation can be probably ascribed to the presence of a few uncoated areas in KHC2000/BG samples where the BG was directly exposed to PBS. Then, the presence of the coating seemed to act as a protection against further weight loss, up to 21 days. Indeed, on day 21, BG and KHC2000/BG scaffolds showed a weight loss of 38.9 ± 8.0% and 22.5 ± 4.1% and a PBS pH value of 10.2 ± 0.1 and 9.9 ± 0.2, respectively. Among the polymer-coated scaffolds, KHC2000E2000-coated BG scaffolds showed the highest percentage of weight loss (45.0 ± 12.6% after 21 days of immersion, significantly higher compared to KHC2000/BG scaffolds (*p <* 0.05)) as well as the highest variability (the longer the samples were soaked in PBS, the higher the standard deviation). Indeed, the inclusion of PEG blocks within the polymer backbone was responsible for the decreased stability of the polymeric coating (see Figure 2A reporting the dissolution/degradation profiles of pure polymeric films incubated in PBS) and, as a consequence, of the KHC2000E2000/BG scaffolds. On the other hand, the high variability in weight loss of KHC2000E2000/BG samples can be correlated with the concurrent occurrence of many different processes of destabilization, namely the dissolution/degradation/detachment of the KHC2000E2000 coating and the degradation of BG.

Furthermore, SEM analyses were carried out on coated and uncoated scaffolds after 21 days immersion in PBS (Figure 10C–F). Whereas crack propagation phenomena were clearly visible on uncoated scaffolds, scaffolds coated with either KHC2000 or PCL did not show any crack. On the other hand, KHC2000E2000/BG scaffolds exhibited several cracks, probably because of the faster degradation/destabilization of this polymer in aqueous medium. Moreover, all the samples exhibited deposition of spherical particles, which were likely amorphous calcium phosphate salts, resulting from the interaction occurring between the phosphate solution and the ions released from the glass-ceramic. According to Fu et al. [66], these particles would transform into crystalline hydroxyapatite with increasing incubation time in PBS.

#### 3.5.4. Mechanical Tests

**Figure 11:**
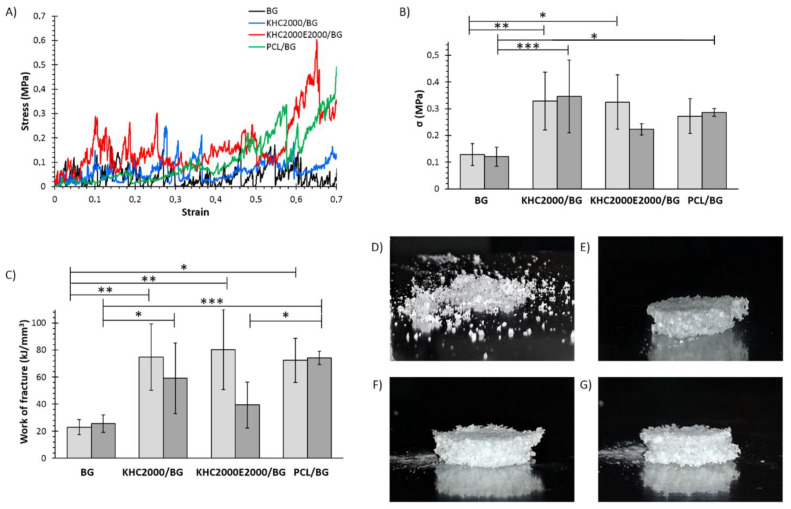
Mechanical characterization of the developed scaffolds. (**A**) Representative compressive stress-strain curves of BG, KHC2000/BG, KHC2000E2000/BG, and PCL/BG scaffolds, (**B**) compressive strength and (**C**) work of fracture of BG, KHC2000/BG, KHC2000E2000/BG, and PCL/BG scaffolds evaluated in dry (light gray) and wet (gray) conditions. The appearance of (**D**) BG, (**E**) PCL/BG, (**F**) KHC2000/BG, and (**G**) KHC2000E2000/BG scaffolds after compression test. * 0.01 < *p* < 0.05, ** 0.001 < *p* < 0.01, and *** 0.0001 < *p* < 0.001.

To investigate the potential of PUR-coated 45S5 BG scaffolds for application in bone tissue engineering, the compressive strength (σ) and the work of fracture of the scaffolds were determined through compressive mechanical tests. As a consequence of their high porosity and hollow structure, all the samples were characterized by a jagged stress-strain curve (Figure 11A), as typical of BG scaffolds obtained with the foam replication technique [20]. However, polymer-coated BG constructs exhibited densification, as highlighted by the fast increase in stress as a function of strain within the range of 60–70%. Moreover, while BG scaffolds appeared completely destroyed at the end of the test due to their very brittle nature, polymer-coated samples maintained their shape (Figure 11D–G), thus further corroborating the occurrence of densification phenomena.

The compressive strength and the work of fracture were measured in dry and wet conditions to better simulate the scaffold’s real working conditions upon implantation (Figure 11B,C). In dry conditions, the compressive strength of BG scaffolds was significantly increased by coating them with the investigated polymers. Since the overall porosity of the samples did not significantly decrease after the coating, this improvement can be exclusively ascribed to the polymeric layer, with no significant contribution due to clogged pores. Moreover, KHC2000/BG scaffolds showed similar compressive strength to KHC2000E2000/BG and PCL/BG samples, although the amount of polymer composing its coating was significantly lower. A similar trend was observed in terms of work of fracture, which significantly increased upon application of the polymeric coating. These results are in agreement with already reported data that correlate the improvement of mechanical properties to polymer capability to fill micropores and microcracks on the surface, similar to collagen in bone [35,37]. Hence, the strengthening and toughening effects exhibited by the here-developed composites could be explained by a mechanism of crack-bridging at the microscale [67,68], which is typical of the fracture behavior of human bones to a certain extent. The obtained mechanical properties fall within the characteristic compressive strength range of spongy bone (0.1–30 MPa) [69]. On the other hand, the concurrent growing of hydroxyapatite and the formation of new tissue are expected to progressively improve the mechanical properties of the scaffolds upon implantation [20]. In the wet state, BG, KHC2000/BG, and PCL/BG scaffolds did not exhibit significant changes in their mechanical properties. On the other hand, mechanical strength and work of fracture of KHC2000E2000/BG samples tended to decrease (no significant differences) in the wet state compared to the dry conditions, as a consequence of KHC2000E2000 higher solubility and destabilization in aqueous media compared to both PCL and KHC2000.

**Figure 12:**
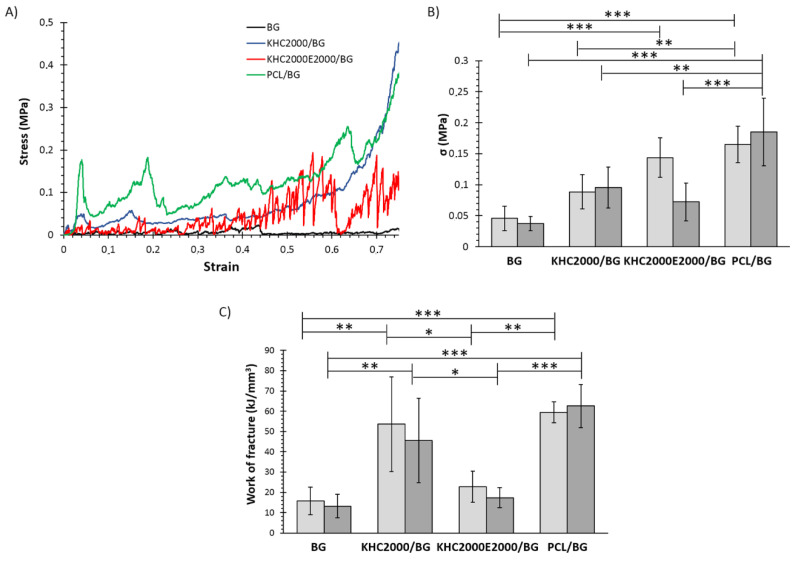
Mechanical characterization of BG, KHC2000/BG, KHC2000E2000/BG, and PCL/BG after 21 days immersion in SBF. (**A**) Representative stress-strain curves of BG, KHC2000/BG, KHC2000E2000/BG, and PCL/BG scaffolds, (**B**) compressive strength and (**C**) work of fracture calculated for each type of scaffold in dry (light gray) and wet (gray) conditions. * 0.01 < *p* < 0.05, ** 0.001 < *p* < 0.01, and *** 0.0001 < *p* < 0.001.

Mechanical tests were also performed on all samples after 3, 7, 14, and 21 days of immersion in SBF, to assess their mechanical behavior during concurrent degradation and HA deposition. The stress-strain curves of the tested samples after 21 days of incubation in SBF are collected in Figure 12A. All the samples showed the typical jagged curves as described before; however, densification phenomena in composite samples occurred earlier, especially for PCL- and KHC2000-coated scaffolds, as a consequence of their higher stability in aqueous media. Figure 12B,C reports the compressive strength and the work of fracture of all types of scaffolds analyzed after 21 days of immersion in SBF, in the dry and wet state. Also after incubation in SBF, KHC2000E2000/BG scaffolds exhibited lower mechanical properties (in particular in terms of mechanical strength) in the wet state compared to dry conditions as a consequence of the higher instability of PEG-containing polymers in aqueous media. As a matter of fact, as a consequence of their lower sensitivity to the surrounding watery environment, this trend was not present in pure BG as well as in samples coated with either PCL or KHC2000.

**Figure 13:**
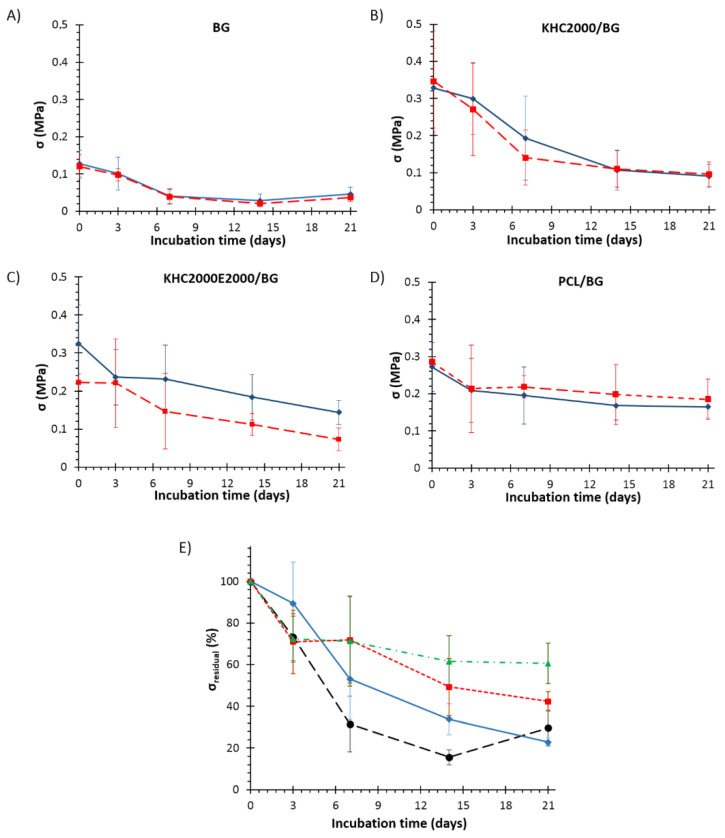
Trend of compressive strength evaluated in dry (blue, continuous line) and wet (red, dashed line) conditions as a function of immersion time in SBF for (**A**) BG, (**B**) KHC2000/BG, (**C**) KHC2000E2000/BG, and (**D**) PCL/BG scaffolds. (**E**) Change in compressive strength (*σ*_residual_ (%)) calculated according to Equation (4), as a function of immersion time in SBF for BG (black, dashed line), PCL/BG (green, dashed-dotted line), KHC2000/BG (blue, continuous line) and KHC2000E2000/BG (red, dashed line) scaffolds.

As expected, after immersion in SBF both compressive strength and work of fracture of the samples decreased due to the dissolution of the BG structure and the conversion to amorphous calcium phosphate [34]. This amorphous layer is responsible for the bioactivity of the samples, but at the same time, it causes the observed decrease in mechanical properties. However, composite scaffolds showed significantly higher mechanical properties compared to BG as such, suggesting that the polymeric coating not only induced an increase in the starting compressive strength but also slowed down the rate of compressive strength decrease during immersion in SBF, as shown in Figure 13A–D, which plot compressive strength against time. Figure 13E, instead, reports the change in compressive strength (*σ*_residual_ (%)) as a function of immersion time in SBF. The compressive strength of BG scaffolds drastically decreased within the first 7 days of immersion, with a loss in compressive strength of approx. 70%. After that, the deterioration rate became much slower and inverted its trend starting from 14 days incubation, probably as a consequence of the progressive crystallization of the hydroxyapatite layer. Differently, PCL-coated scaffolds seemed to better retain their mechanical strength, with a decrease of approx. 30% after 3 days of incubation in SBF, which increased to circa 40% on day 21. KHC2000/BG scaffolds initially showed the lowest decrease of compressive strength compared to the other samples (compressive strength was 0.30 ± 0.09 MPa, almost 90% of the initial value after 3 days immersion in aqueous medium). However, starting from 3 days incubation in SBF, KHC2000/BG scaffolds started to progressively lose their mechanical properties reaching a final mechanical strength of ca. 0.1 MPa after 3 weeks immersion in SBF. In agreement with its higher instability in aqueous media, KHC2000E20000/BG scaffolds showed an almost linear decrease in mechanical strength with increasing incubation time in SBF.

The decrease in the rate of mechanical properties deterioration observed in PCL/BG scaffolds can be correlated with the progressive deposition of the HA layer that tended to mitigate the effects of BG degradation, in a similar way as in pure BG structures. This behavior was made possible by the presence of irregularities in the PCL coating, which favored the nucleation of HA, as also proved by XRD analyses. Similarly, in KHC2000E2000/BG scaffolds, the capability to retain approx. the 40% of their initial mechanical strength can be correlated to HA deposition, which was made possible by the high instability of this polymeric coating in aqueous media, although the process of HA nucleation was slower compared to pure BG. Conversely, in the case of KHC2000/BG the strengthening effect coming from hydroxyapatite deposition was not observed within the investigated time interval because the more homogeneous coating of these structures made the process of HA nucleation slower compared to the other designed composite scaffolds. Nevertheless, KHC2000/BG samples better retained their native compressive strength (i.e., higher *σ*_residual_) compared to BG scaffolds at each investigated time point. After 21 days of incubation in SBF, KHC2000/BG and BG samples showed similar *σ*_residual_ values despite the delayed hydroxyapatite deposition of KHC2000-coated scaffolds.

#### 3.5.5. Biological Tests

**Figure 14:**
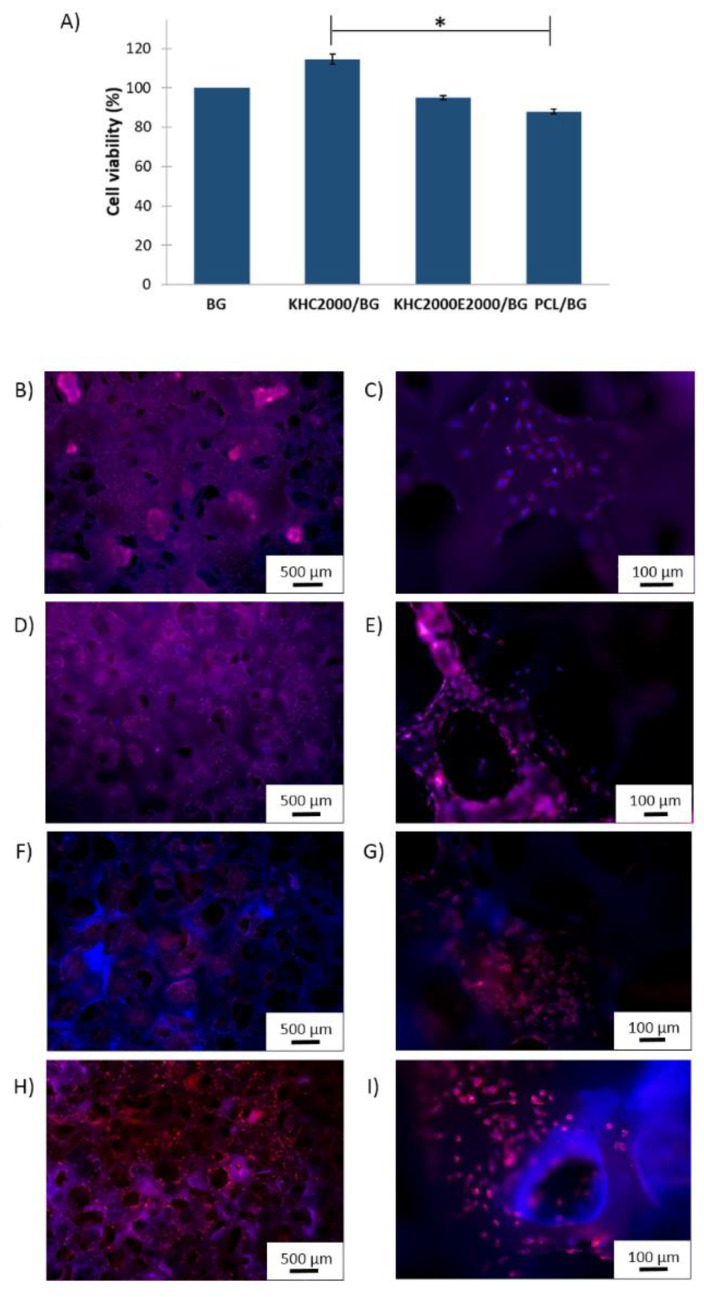
(**A**) Cell viability evaluated using WST-8 assay (BG scaffolds were used as control samples). Fluorescent images of (**B**,**C**) BG; (**D**,**E**) PCL/BG; (**F**,**G**) KHC2000/BG and (**H**,**I**) KHC2000E2000/BG scaffolds. Cell actin filaments and nuclei were stained with Rhodamine Phalloidin (red) and DAPI (blue), respectively.

MG-63 osteoblast-like cells were seeded on pure and polymer-coated BG scaffolds and cultivated for 2 days to conduct a primary investigation of the cytocompatibility of the designed composite scaffolds and their potential application for BTE. Cell viability was investigated through WST-8 assay, to obtain a quantitative analysis of the mitochondrial activity of viable cells seeded into the scaffolds. Cell viability was evaluated using BG scaffolds as control samples as large evidence of their ability to enhance adhesion, growth, and differentiation of osteoblasts has been reported in the literature [70,71,72,73,74]. All the composites resulted to be cytocompatible with no significant differences in cell viability between coated and uncoated scaffolds (Figure 14A). However, the relative mitochondrial activity of cells seeded into KHC2000-coated BG scaffolds was significantly (*p* < 0.05) higher than that assessed on PCL composite scaffolds, suggesting an improvement in cell behavior in PUR-coated scaffolds with respect to samples coated with commercially available poly(ε-caprolactone). To better investigate cell viability and morphology, fluorescence analyses were carried out. Figure 14B-I reports fluorescence images of cells adhered to the scaffolds with cell cytoskeleton and nuclei colored in red and blue, respectively. All the scaffolds were highly colonized by cells also in their inner cavities, thanks to their highly porous and interconnected structure [74].

## 4. Conclusions

In this work, poly(urethane) biomaterials have been proposed as coating materials of porous 45S5 BG-based scaffolds to improve their mechanical performance without inhibiting their characteristic bioactive behavior. Two PURs differing in the composition of their soft segment (poly(ε-caprolactone) or poly(ε-caprolactone)/poly(ethylene glycol) 70/30 *w*/*w*) (KHC2000 and KHC2000E2000, respectively) were synthesized to this purpose and thoroughly characterized in terms of physico-chemical, superficial, and mechanical properties. A protocol for the coating of the scaffolds with both polymers was thus optimized to achieve homogeneous BG structure covering while maintaining high porosity. PCL-coated scaffolds were also fabricated and characterized as control samples. The use of KHC2000 led to highly porous KHC2000/BG structures and the homogenous and adherent coating did not inhibit BG bioactivity. Moreover, these scaffolds exhibited significantly improved compressive strength compared to BG scaffolds as such, although the amount of polymer forming the coating was much lower compared to the other investigated samples (i.e., KHC2000E2000/BG and PCL/BG). The coating provided well-maintained mechanical properties after several days in SBF, although a slight deterioration after a long (15 days) immersion period was observed. Scaffolds coated with KHC2000E2000 presented homogenous coating and bioactive behavior as well as improved mechanical properties in dry conditions compared to BG scaffolds as such. However, the presence of PEG in the PUR backbone conferred instability in aqueous environment and the mechanical reliability drastically decreased in wet conditions. Irrespective of the coating material, all the developed matrices exhibited high biocompatibility. PCL-based PURs could thus represent a promising alternative to commercial PCL as coating materials for BG scaffolds. In particular, KHC2000 exhibited better adherence and a more uniform coating to BG compared to PCL and allowed the achievement of improved mechanical properties with a thinner coating. Moreover, the use of this polymer as BG coating resulted in composite scaffolds with improved stability in a watery environment and proper mechanical properties for bone tissue engineering applications in wet conditions. With this material, a further improvement in mechanical properties could probably be achieved by increasing the thickness of the coating through multiple dipping procedures.

Altogether, the results of this work have proven the potential of tailor-made PURs as coating materials for 45S5 BG-based scaffolds, with the additional key feature of allowing *ad-hoc* customization of their building block composition, which results in the possibility to finely tune degradation kinetics, mechanical properties, and biomimetic properties. For instance, coating degradation kinetics could be modulated by incorporating PEG (as demonstrated in this work) or enzyme-sensitive moieties (e.g., the Ala-Ala motif) into the PUR chains [42]. Conversely, the biomimetic properties of the coating material could be improved through PUR bulk functionalization with adhesive and bioactive peptide sequences [75]. Finally, the presence of N-H groups within the poly(urethane urea) backbone resulted in a highly adherent coating which was not achieved using PCL.

## Figures and Tables

**Table 1:** Density of the polymers, $\rho_{\mathrm{coat}}$.

Polymer	$\rho_{\mathrm{coat}}\;(g/{\mathrm{cm}}^{3})$
PCL	1.145 [34]
KHC2000	1.1 ± 0.04
KHC2000E2000	1.07 ± 0.03

**Table 2:** Mechanical properties of PCL, KHC2000, and KHC2000E2000.

	Young’s Modulus (MPa)	Stress at Break (MPa)	Strain at Break (%)
PCL	210.3 ± 28.8	26.8 ± 4.0	683.3 ± 9.7
KHC2000	13.8 ± 1.0	20.3 ± 3.1	822.5 ± 79.7
KHC2000E2000	11.8 ± 2.6	11.2 ± 0.7	1145.0 ± 117.4

**Table 3:** Average dimensions of pure BG scaffolds.

Parameter	
Weight	0.052 ± 0.01 g
Diameter	7.8 ± 0.2 mm
Height	5.1 ± 0.1 mm
Porosity	92.1 ± 1.6%
Pore size	200 ÷ 600 μm

**Table 4:** The average amount of polymer covering the composites and porosity after coating.

	Amount of Polymer in the Coating (mg)	Porosity (%)
PCL/BG	1.5 ± 0.3	90.7 ± 1.6
KHC2000/BG	0.9 ± 0.2	91.1 ± 1.7
KHC2000E2000/BG	1.5 ± 0.5	91.7 ± 1.5

## Data Availability

The data presented in this study are available on request from the corresponding author.

## Supplementary Materials

The following are available online at https://www.mdpi.com/article/10.3390/polym14010151/s1, Figure S1: Chemical structures of PUR building blocks. Figure S2: Scheme of the protocol adopted for poly(urethane) synthesis. Table S1: Reagents required to prepare 1 L of SBF. Figure S3: ATR-FTIR spectra of macrodiols and as synthesized poly(urethane)s. Figure S4: Profile of deposited water drop on polymer films at different time points. Figure S5: Normalized RID signal as a function of retention time during polymer degradation. Figure S6: Pore size distribution in BG scaffold. Figure S7: ATR-FTIR and XRD spectra of BG scaffold after immersion in SBF for different time intervals.
