# Supplementary material for: Custom-Made Poly(urethane) Coatings Improve the Mechanical Properties of Bioactive Glass Scaffolds Designed for Bone Tissue Engineering
Source: Polymers (Basel). 2021 Dec 31;14(1):151. doi: 10.3390/polym14010151 (PMC8747464; doi:10.3390/polym14010151)
Supplement: Supplementary file 1 [file polymers-14-00151-s001.zip › polymers-1465646-supplementary.pdf]

Custom-made Poly(urethane) Coatings Improve the Mechanical Properties of Bioactive Glass Scaffolds Designed for Bone Tissue Engineering

Monica Boffito [1,*], Lucia Servello [1], Marcela Arango-Ospina [2], Serena Miglietta [1], Martina Tortorici [1,3†], Susanna Sartori [1], Gianluca Ciardelli [1,‡] and Aldo R. Boccaccini [2,*,‡]

[1] Department of Mechanical and Aerospace Engineering, Politecnico di Torino, Corso Duca degli Abruzzi 24, 10129 Turin, Italy. lucia.servello@polito.it (L.S.); serena.miglietta@studenti.polito.it (S.M.); martina.tortorici@outlook.com (M.T.); susanna.sartori@polito.it (S.S.); gianluca.ciardelli@polito.it (G.C.)
[2] Institute of Biomaterials, University of Erlangen-Nuremberg, Cauerstr. 6, 91058 Erlangen, Germany. marcela.arango@fau.de (M.A.O.)
[3] Julius Wolff Institut, Charité - Universitätsmedizin Berlin, Augustenburger Platz 1, 13353 Berlin, Germany.
† Current affiliation: Julius Wolff Institut, Charité - Universitätsmedizin Berlin, Augustenburger Platz 1, 13353 Berlin, Germany.
‡ G. C. and A.R. Boccaccini share last authorship
* Correspondence: monica.boffito@polito.it (M.B.); aldo.boccaccini@ww.uni-erlangen.de (A.R.B.)

1. Poly(urethane) Synthesis

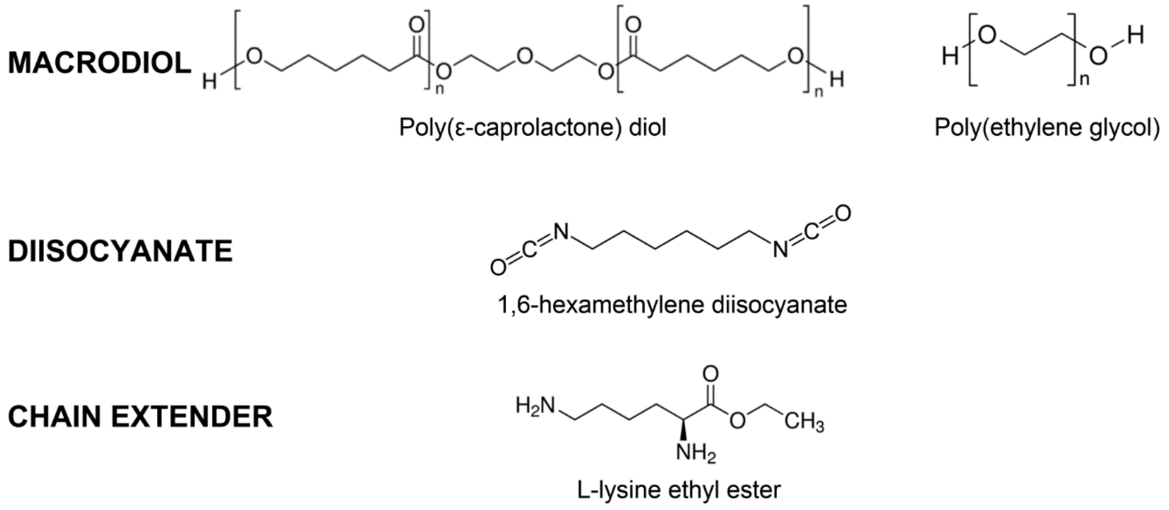

Figure S1. Chemical structures of the reagents used for poly(urethane) synthesis.

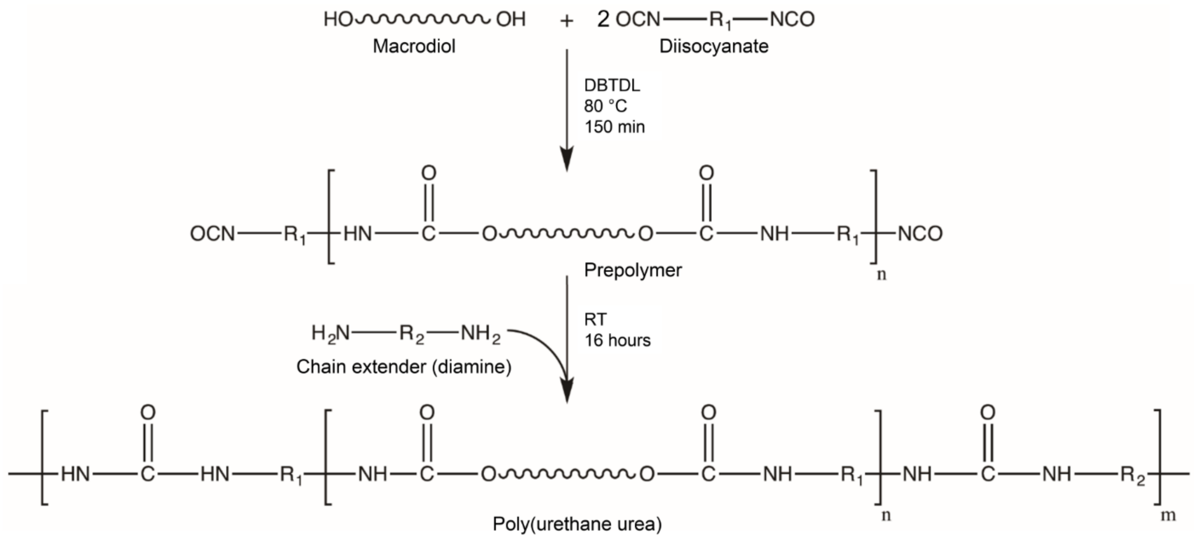

Figure S2. Scheme of the protocol adopted for poly(urethane urea) synthesis. The macrodiol (PCL diol or a mixture of PCL diol and PEG) was reacted with the diisocyanate HDI (2:1 molar ratio with respect to the macrodiol) in the presence of the catalyst DBTDL at 80 °C for 2.5 hours. Then the chain extender (L-Lysine ethyl ester dihydrochloride neutralized with triethylamine, chain extender at 1:1 molar ratio with respect to the macrodiol) was added and the chain extension reaction occurred at room temperature for 16 hours.

2. Preparation Protocol of Simulated Body Fluid (SBF)

Simulated body fluid (SBF) was prepared according to the protocol developed by Kokubo et al. [1]. Briefly, to prepare 1000 mL of SBF, 700 mL of double demineralized water (ddH_2O) were first heated at 36.5 ± 1.5 °C in a plastic bottle. Then, the following chemicals were required: sodium chloride (NaCl), sodium hydrogen carbonate ($NaHCO_3$), potassium chloride (KCl), di-potassium hydrogen phosphate trihydrate ($K_2PO_4{\cdot}3H_2O$), magnesium chloride hexahydrate ($MgCl_2{\cdot}6H_2O$), calcium chloride dihydrate ($CaCl_2{\cdot}2H_2O$), sodium sulfate (Na_2SO_4), tris-hydroxymethyl aminomethane ($(HOCH_2)_3CNH_2$) (Tris) and 1.0 M hydrochloric acid (1M-HCl). Chemicals were added according to the order and in the amounts reported in Supplementary Table S1. Initially, the first 7 chemicals were dissolved; then ddH_2O was raised up to 900 mL and Tris was added slowly spoon by spoon avoiding pH increase at values higher than 7.45. Every time the pH reached 7.45, 1M-HCl was added to decrease it to 7.42 ± 0.01. Tris and 1M-HCl were thus added alternately to control the pH until the total amount of Tris was dissolved. Then, the pH was adjusted at 7.40 while keeping the solution at 36.5 °C and ddH_2O was added up to 1000 mL volume. Finally, the SBF solution was equilibrated at room temperature and stored at 4 °C. SBF was again equilibrated at 37 °C before use in the bioactivity tests.

Table S1. Reagents required to prepare 1000 mL of SBF

Order	Chemical	Amount	Purity (%)	Supplier
1	NaCl	7.9948g	100	VWR Chemicals, Germany
2	$NaHCO_3$	0.3532 g	100	Sigma Aldrich, Germany
3	KCl	0.225 g	99.5	Merck KGaA, Germany
4	$K_2PO_4{\cdot}3H_2O$	0.231 g	99.0	Sigma Aldrich, Japan
5	$MgCl_2{\cdot}6H_2O$	0.3048 g	100	Sigma Aldrich, Japan
6	1M-HCl	39 mL		VWR Chemicals, France
7	$CaCl_2{\cdot}2H_2O$	0.3638 g	100	VWR Chemicals, France
8	Na_2SO_4	0.0719 g	99.2	VWR Chemicals, France
9	Tris	6.0568 g	100	VWR Chemicals, Belgium
10	1M-HCl			VWR Chemicals, France

3. Poly(urethane) Characterization

3.1. Attenuated Total Reflectance Fourier Transform Infrared (ATR-FTIR) Spectroscopy

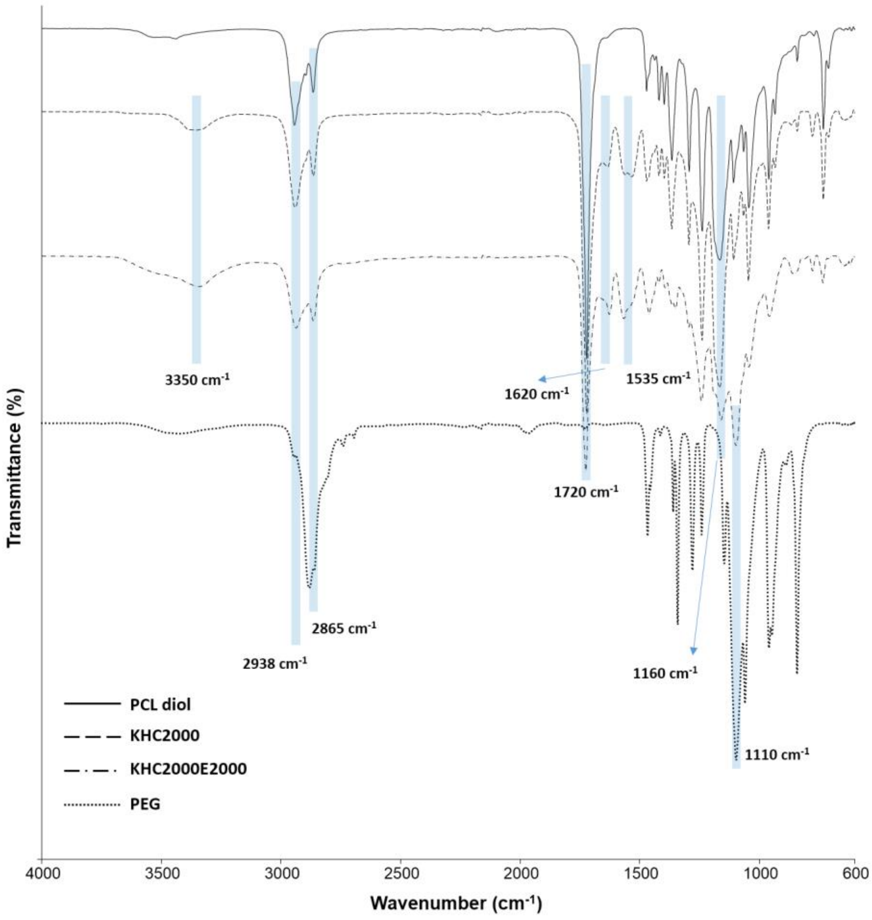

Figure S3. ATR-FTIR spectra of (from top to bottom) PCL diol (black, continuous line), KHC2000 (black, dashed line), KHC2000E2000 (black, dashed-dotted line) and PEG (black, dotted line). The characteristic peaks of newly formed urethane/urea bonds, PCL and PEG are highlighted.

3.2. Contact Angle Measurements

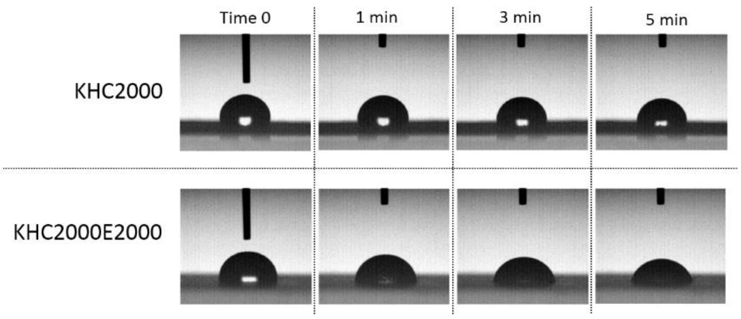

Figure S4. Profile of a water drop deposited on KHC2000 and KHC2000E2000 films at different time points (immediately after deposition and 1, 3 and 5 minutes after deposition).

3.3. *Size Exclusion Chromatography (SEC)*

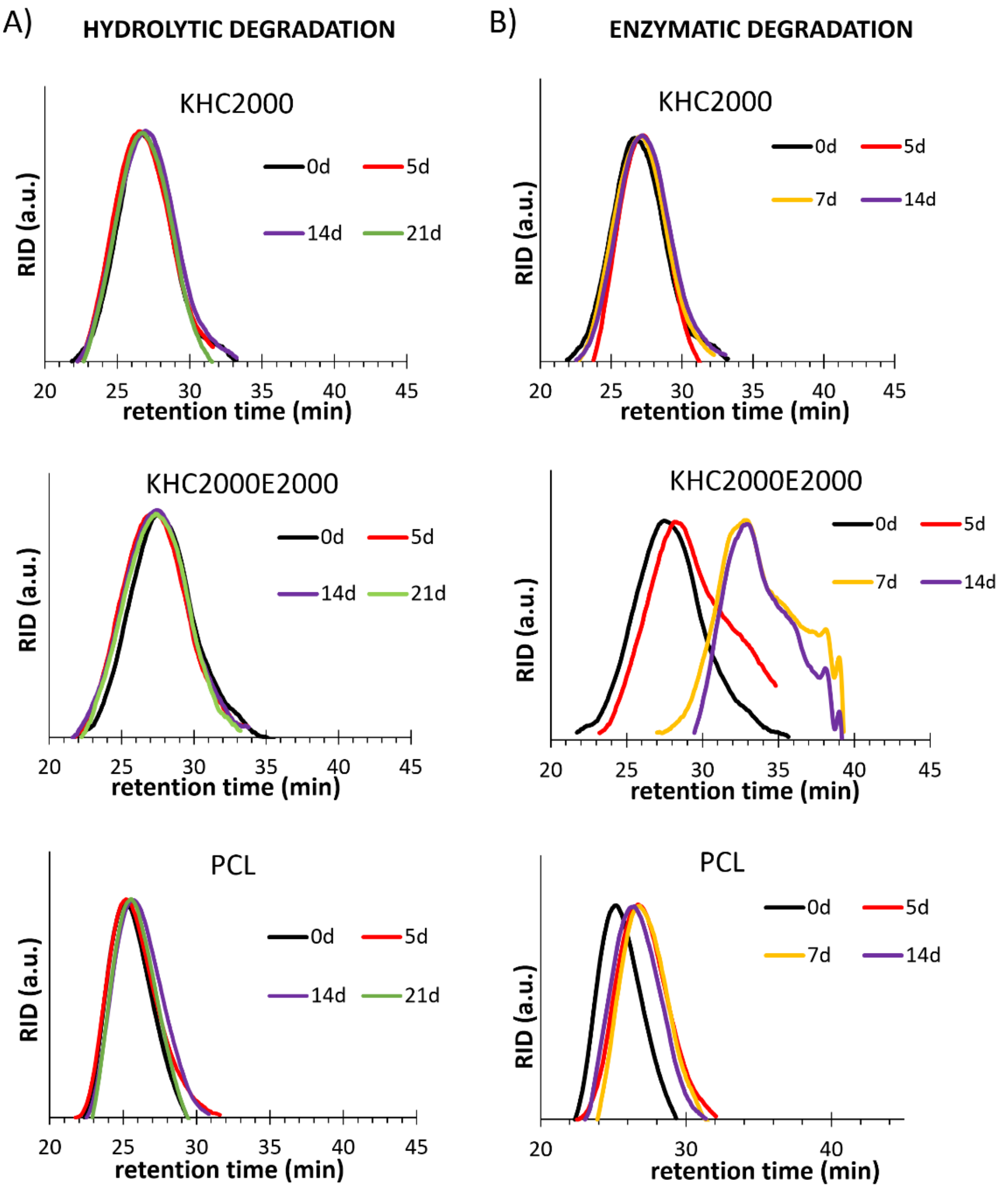

Figure S5. Normalized RID signal as a function of retention time for KHC2000, KHC2000E2000 and PCL at different time points registered during hydrolytic (**A**) and enzymatic (**B**) degradation at 37 °C.

4. Scaffold Characterization

4.1. Scaffold Pore Size

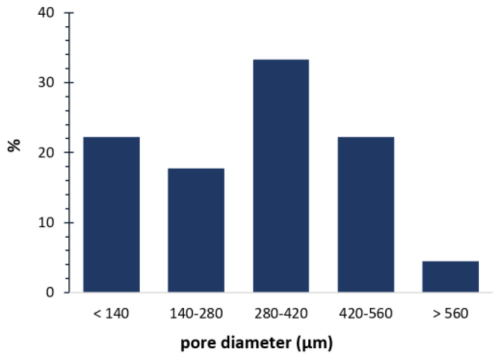

Figure S6. Pore size distribution in a pure BG scaffold.

4.2. Bioactivity Tests on BG Scaffolds

Supplementary Figure S7A reports an overall view of the modifications of BG ATR-FTIR spectrum with increasing incubation time in SBF. In the spectrum of native BG (0d), the peak in the range 1100-1000 cm^{-1} can be ascribed to the concomitant P=O and Si-O-Si asymmetric stretching [2], meanwhile the Si-O bond (non-bridging oxygen) shows absorption at 915 cm^{-1}. The other BG characteristic peaks appear at 438 cm^{-1} due to the Si-O-Si bending vibration, and at 619, 575 and 521 cm^{-1} attributed to P-O bending that suggests the presence of a calcium phosphate phase [3]. Soaking the samples in SBF led to the appearance of new peaks which can be ascribed to the growing of a HCA layer: after 3 days incubation in SBF, a new peak at *ca.* 800 cm^{-1} appeared (Si-O-Si stretching vibration) as a consequence of the formation of a silica rich layer [4]. Furthermore, CO_3^{2-} groups in the carbonate HA layer generated a peak at 1418 cm^{-1} due to C=O stretching. The double peak at *ca.* 570 cm^{-1} can be ascribed to P-O bending vibration indicating the crystallization of calcium phosphate into HCA [5]. In addition, the peak due to Si-O stretching progressively disappeared and the residual peak at 1100 cm^{-1} resulted only from P=O stretching. Moreover, also the peaks in the region 619-575 cm^{-1} due to P-O bending vibration gradually disappeared proving the dynamical transformation of the surface and its degradation. Finally, the peak at 438 cm^{-1} was slightly shifted probably because of the variation of ionic concentration [4].
Figure S7B reports the XRD spectra of uncoated BG scaffolds upon immersion in SBF for 0, 1, 3, 7, 14, and 21 days. XRD spectrum of native BG (0d) exhibited the major peaks at 20°, 24°, 27°, 34°, 49°, and 60°. As expected, the sintering process of 45S5 Bioglass® scaffolds led to the formation of $Na_2Ca_2Si_3O_9$ (i.e., combeite) as main crystalline phase [6]. Moreover, XRD analyses performed upon sample soaking in SBF confirmed that the HA layer visible in SEM images (Figure 9A of the manuscript) was not only composed of amorphous calcium phosphate, but also of crystalline hydroxyapatite. During immersion in SBF, the characteristic peaks of combeite gradually disappeared, meanwhile a new peak at $2\theta=32°$ became narrow. Finally, after 21 days of immersion in SBF, the XRD spectrum of BG scaffold reported the typical trend of an amorphous phase, with the addition of two peaks at 26° and 32° that are related to the deposition of crystalline hydroxyapatite [7]. Hence, in addition to the deposition of crystalline HA, a decrease in crystallinity occurred during incubation in SBF as a consequence of combeite progressive degradation [6].

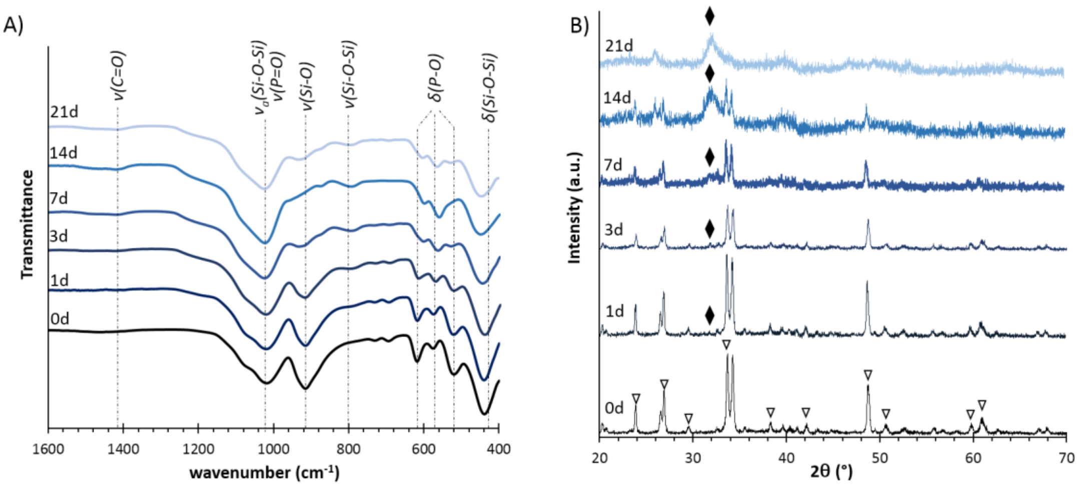

Figure S7. ATR-FTIR (**A**) and XRD (**B**) spectra of 45S5 Bioglass®-based scaffold after immersion in SBF for different time intervals (0, 1, 3, 7, 14, and 21 days).

5. References

1. Kokubo, T.; Takadama, H. How useful is SBF in predicting in vivo bone bioactivity? *Biomaterials* **2006**, *27*, 2907–2915.
2. Filho, O.P.; La Torre, G.P.; Hench, L.L. Effect of crystallization on apatite-layer formation of bioactive glass 45S5. *J. Biomed. Mater. Res.* **1996**, *30*, 509-514.
3. Lefebvre, L.; Chevalier, J.; Gremillard, L.; Zenati, R.; Thollet, G.; Bernache-Assolant, D.; Govin, A. Structural transformations of bioactive glass 45S5 with thermal treatments. *Acta Mater.* **2007**, *55*, 3305–3313.
4. Cerruti, M.; Greenspan, D.; Powers, K. Effect of pH and ionic strength on the reactivity of Bioglass® 45S5. *Biomaterials* **2005**, *26*, 1665–1674.
5. Wu, Z.Y.; Hill, R.G.; Yue, S.; Nightingale, D.; Lee, P.D.; Jones, J.R. Melt-derived bioactive glass scaffolds produced by a gel-cast foaming technique. *Acta Biomater.* **2011**, *7*, 1807–1816.
6. Chen, Q.Z.; Thompson, I.D.; Boccaccini, A.R. 45S5 Bioglass®-derived glass–ceramic scaffolds for bone tissue engineering. *Biomaterials* **2006**, *27*, 2414–2425.
7. Lin, K.S.K.; Tseng, Y.H.; Mou, Y.; Hsu, Y.C.; Yang, C.M.; Chan, J.C.C. Mechanistic study of apatite formation on bioactive glass surface using 31P solid-state NMR spectroscopy. *Chem. Mater.* **2005**, *17*, 4493–4501.
